# Adherence Reporting in Randomized Controlled Trials Examining Manualized Multisession Online Interventions: Systematic Review of Practices and Proposal for Reporting Standards

**DOI:** 10.2196/14181

**Published:** 2019-08-15

**Authors:** Ina Beintner, Bianka Vollert, Anna-Carlotta Zarski, Felix Bolinski, Peter Musiat, Dennis Görlich, David Daniel Ebert, Corinna Jacobi

**Affiliations:** 1 Faculty of Psychology School of Science Technische Universität Dresden Dresden Germany; 2 Institute of Psychology Faculty of Humanities, Social Sciences, and Theology Friedrich-Alexander-University Erlangen-Nürnberg Erlangen Germany; 3 Department of Clinical, Neuro- and Developmental Psychology Faculty of Behavioural and Movement Sciences Vrije Universiteit Amsterdam Amsterdam Netherlands; 4 Department of Psychological Medicine Institute of Psychiatry, Psychology and Neuroscience King’s College London London United Kingdom; 5 Institute of Biostatistics and Clinical Research Faculty of Medicine Westfälische Wilhelms-Universität Münster Münster Germany

**Keywords:** adherence, compliance, usage, attrition, ehealth, e-mental health, mental health, behavior change, reporting standards, CONSORT eHealth, review

## Abstract

**Background:**

Adherence reflects the extent to which individuals experience or engage with the content of online interventions and poses a major challenge. Neglecting to examine and report adherence and its relation to outcomes can compromise the interpretation of research findings.

**Objective:**

The aim of this systematic review is to analyze how adherence is accounted for in publications and to propose standards for measuring and reporting adherence to online interventions.

**Methods:**

We performed a systematic review of randomized controlled trials on online interventions for the prevention and treatment of common mental disorders (depression, anxiety disorders, substance related disorders, and eating disorders) published between January 2006 and May 2018 and indexed in Medline and Web of Science. We included primary publications on manualized online treatments (more than 1 session and successive access to content) and examined how adherence was reported in these publications.

**Results:**

We identified 216 publications that met our inclusion criteria. Adherence was addressed in 85% of full-text manuscripts, but only in 31% of abstracts. A median of three usage metrics were reported; the most frequently reported usage metric (61%) was intervention completion. Manuscripts published in specialized electronic health journals more frequently included information on the relation of adherence and outcomes.

**Conclusions:**

We found substantial variety in the reporting of adherence and the usage metrics used to operationalize adherence. This limits the comparability of results and impedes the integration of findings from different studies. Based on our findings, we propose reporting standards for future publications on online interventions.

## Introduction

Online interventions have become popular in the prevention and treatment of mental disorders, and they have been shown to be effective in clinical trials for a wide range of common mental disorders [1-6]. These interventions typically include multiple interactive self-help lessons to improve mental health by using established psychotherapeutic techniques. These lessons can be delivered via a Web browser or mobile app [7].

However, the behavior changes that online interventions aim to induce are very unlikely to occur if participants expose themselves to the intervention only briefly or do not do so at all. Adherence can be conceptualized as the extent to which individuals experience or engage with the content of an online intervention [8]. Poor adherence is a major issue in almost all these interventions [9] and even more so if they are unguided [10].

Attention to adherence has increased over time. However, even 13 years after Eysenbach’s landmark paper “The law of attrition” [11], referring to the finding that a significant proportion of participants in electronic health (eHealth) research do not fully use the studied technology, adherence is still not consistently and systematically examined and reported in studies of online interventions. Additionally, operationalization of adherence varies substantially across trials [12], limiting the comparability of results between trials. However, neglecting to examine adherence and its impact on outcomes in online intervention trials can compromise the interpretation of research findings, and, in turn, lead to inappropriate recommendations and decisions regarding the use and implementation of such interventions.

If an intervention is not effective even though the participants have been using it the way they were supposed to, it is very likely that the intervention itself is not working and that the core components of the intervention need to be changed or that there is a mismatch between user needs and intervention components. If, however, the intervention is not effective while people are not exposing themselves to a sufficient “dose” of it, implications for further research might be quite different. A mismatch between user needs and the intervention or its components is likely in these cases. Poor adherence may then lead to systematic underestimation of the potential intervention effects. Instead of changing core components of the intervention that teach skills and prompt change in behaviors related to mental health, the intervention may need to be augmented with components to improve adherence, or recruitment strategies may need to be changed to reach those who are open to actually using the interventions. Thus, it is vital to study both intervention effects of and adherence to online interventions and their interactions simultaneously. Furthermore, it is important to identify differential usage patterns in multicomponent interventions and user characteristics that are associated with these patterns [13-15]. In order to achieve this, multiple usage metrics are needed [15].

Although adherence has received increasing attention in the study of online interventions and been addressed in existing reporting guidelines [16], the field is still lacking common standards for addressing and reporting adherence. Various terms, definitions, and measures have been used to describe how users engage with online interventions. Some terms have been adopted from the field of pharmacotherapy [17] and others, from guidelines to describe participant flow in clinical research trials [16,18]. Although the term adherence is widely used, some authors also use the terms compliance, (session) attendance, engagement, user retention, persistence, exposure, intervention usage, or polar opposite terms—attrition or (treatment or study) dropout. Even when authors use the same term, they do not necessarily mean the same thing. For example, the term dropout can either refer to the premature cessation of treatment (treatment dropout) or the noncompletion of postintervention assessments (study dropout), although some investigators equate both [19]. In a similar way, the term attrition is used to refer to the loss of participants in the intervention of a (clinical or epidemiological) trial. For trials examining online interventions, it has been proposed that “nonusage attrition” (comparable to treatment dropout) and “dropout attrition” (comparable to study dropout) should be distinguished from each other, and it has been postulated that these forms of attrition are related to each other [11]. For this review, we chose the term adherence as an umbrella term for describing how participants use online interventions, because this term implies that they have to actively engage with an intervention [20]; the term can also be used outside of clinical trials.

In addition to a variety of terms that describe how users adhere to interventions, there are also numerous ways to measure adherence. Most online intervention platforms store log data (eg, time spent on the intervention page), which allow us to track if and how users interact with the intervention. Despite some shortcomings of this data collection method, such as not knowing with certainty whether the person who used the program was the same person who signed up, or whether a user actually engaged with the content or just opened the pages without further engagement, it provides us with objective and comparable usage metrics. However, there is much variety in the usage metrics reported for online interventions (eg, percentage of participants completing all modules/sessions, percentage of participants who visited/revisited websites, average duration of visits, average number of log-ins, and average number of pages visited) [12,21]. In addition, usage related to specific program components can only be reported for interventions that include the component (eg, a discussion board or diaries). In addition, some usage metrics only apply to guided interventions (eg, the number of messages sent to a coach). Thus, the number and type of appropriate usage metrics clearly depend on the design and delivery mode of an intervention [9,22]. In addition, the way usage is tracked and stored has an impact on which usage data and types of adherence are available afterward. In a recent review on the concept of adherence to health technology, Sieverink et al [12] brought up an additional aspect: In order to define adherence, the intended use of an intervention would have to be both defined and justified by the developer (comparable to the optimum dose of a medication) beforehand. However, we still know very little about the necessary dose of online interventions to achieve optimal outcomes. The authors of the review point out that, all too often, developers of online interventions implicitly assume that their interventions work best if all users expose themselves to all parts of the content, and other patterns of use are rarely considered.

Michie et al [15] have pointed out that engagement with online behavior change interventions is complex, depends on the intervention context, and is not limited to technology usage (adherence; micro level of engagement) but extends to behavior change outside the intervention (macro level of engagement). It has been argued that usage metrics such as the completion of exercises, homework, or diaries (where an active input from the user is required) might be linked more closely to intervention outcome rather than measures reflecting passive consumption of content [14]. Macro level engagement is very specific to the behavior change intended with an intervention and likely more complex than engagement at the micro level. Thus, quantitative measures of macro level engagement will always be specific to the type of intervention. Quantitative measures for micro level engagement or adherence on the other hand reflect the structure of an intervention rather than its content and goals. They can therefore be harmonized across interventions. These measures can also be utilized to identify usage patterns.

Although higher adherence has been shown to be linked to larger intervention effects in numerous trials [23-28], other trials [29-33] found no impact of adherence on outcomes. Heterogeneous methods for measuring and reporting adherence as well as examining the dose-response relationship between adherence and outcome and neglecting to consider differential usage patterns may contribute to these conflicting findings.

The CONSORT-eHEALTH (Consolidated Standards of Reporting Trials of Electronic and Mobile HEalth Applications and onLine TeleHealth) guidelines [16] offer recommendations on how to report usage data for online interventions. It is worth noting that in these guidelines, splitting up reports of research findings from one trial into several publications (eg, main outcomes and adherence) is explicitly discouraged (“salami publication”), and the reporting of information on usage is expected in primary publications. Furthermore, CONSORT-eHEALTH highly recommends to report usage metrics both in the abstract and the results sections and to describe usage parameters in the methods section, including details on what recommendations and instructions were given to the users. Moreover, subgroup analyses including only participants who used the intervention are highly recommended. Discussing bias due to nonusage is considered essential. However, there are no precise recommendations on which usage metrics should be chosen for different types of online interventions (eg, guided vs unguided, single vs multisession), because there was no consensus at the time CONSORT-eHEALTH was first published.

The aim of our systematic review was to analyze how adherence has been addressed and which usage metrics have been reported in primary publications on randomized controlled trials (RCTs) evaluating manualized, multisession online interventions (including interventions that are delivered through mobile devices) for common mental disorders (ie, depression, anxiety, substance use disorders, and eating disorders). Specifically, we examined whether adherence was reported in the abstract, the results section, and the CONSORT flowchart of each publication; which usage metrics were reported; whether usage or adherence were addressed in the discussion section; and whether usage metrics were analyzed in relation to outcome.

Based on our findings, we propose common standards for addressing adherence, including specific recommendations for usage metrics that the existing CONSORT-eHEALTH guidelines are currently not specifying.

## Methods

### Inclusion and Exclusion Criteria

We included articles that met the following criteria in the review: (1) the article was published in a peer reviewed journal after the publication of Eysenbach’s seminal “The Law of attrition” [11], between January 2006 and May 22, 2018; (2) the article reported research on an online intervention targeting a common mental disorder (depression [without bipolar disorder, postpartum depression, and complicated grief], anxiety disorders [without posttraumatic stress disorder and obsessive-compulsive disorder], substance use disorders, and eating disorders); (3) the article reported the main findings from an RCT; (4) the trial examined a manualized, multisession (two or more) online intervention (Web- or app-based); (5) participants received sequential access to the intervention content; (6) the intervention taught the participants skills; and (7) the article was written in English or German.

We excluded articles that met the following criteria from the review: (1) The article described research on an online intervention targeting common mental disorders in patients with a primary somatic disorder (eg, diabetes or cancer) or an online intervention targeted at carers or parents of patients; (2) the trial examined a highly individualized intervention without common core content; (3) the trial examined a blended intervention, and (4) the trial examined an intervention purely based on text messaging, email, online discussion boards, or online chat groups.

This review has not been preregistered, and the review protocol has not been published.

### Search Strategy

We conducted a literature search using the Medline and Web of Science databases. We used the following search terms: “online,” “internet,” “webbased,” “mobile”; “treatment,” “psychotherapy,” “therapy,” “self-help,” “prevention,” “intervention”; and “depression,” “depressive,” “anxiety,” “phobia,” “phobic,” “eating disorder,” “disordered eating,” “anorexia,” “anorexic,” “bulimia,” “bulimic,” “binge eating,” “substance abuse,” “substance related disorder,” “alcohol,” “nicotine,” and “cannabis” (Multimedia Appendix 1).

### Study Selection

Studies were selected in two steps. First, titles and abstracts were screened by author IB to exclude publications that were clearly out of the scope of the review; did not report studies on interventions targeting common mental disorders; described study protocols, reviews, and meta analyses; reported secondary analyses only; or had not been published in a peer review journal. Second, authors IB, BV, PM, and AZ assessed the remaining full-text articles for eligibility. Each publication was assessed by two authors. We coded the following variables (along the bibliographic data) for each publication: (1) Is this an RCT? (2) Is this an online intervention? (3) Is it manualized? (4) Does it have multiple sequential sessions/modules? (5) Does it teach skills? (6) Is this the main publication? (7) Study registration number.

### Data Extraction

Data extraction was conducted in three steps. First, authors BV and IB coded the following variables for all included studies: (1) Is adherence addressed in the abstract? (2) Is adherence addressed in the results section? (3) Is adherence addressed in the CONSORT statement? (4) Is adherence examined in relation to outcome? (5) Is adherence addressed in the discussion section?

In the next step, authors BV and IB extracted the information on adherence reported in the results section and the CONSORT statement. For that purpose, a data extraction form was developed, which captured the following usage metrics: full intervention completion (eg, “XX%/N completed the full intervention” or “XX%/N failed to complete the full intervention”), completion of a set minimum of sessions/modules (eg, “XX%/N completed 6 out of 8 sessions” or “XX%/N completed less than 2 out of 5 sessions”), average number of completed sessions/modules, specified point of intervention dropout (last opened session or module, sometimes illustrated by a graph), intervention dropout (not specified, eg, “XX%/N did not complete the intervention”), number of participants who were allocated to the intervention but never logged on, number of times participants logged on, proportion of patients accessing the treatment site per week, total time spent on the program, time spent on the program site per week/per login, and number of participants who completed a survey that is part of the intervention (not just assessment for the clinical trial), number of entries in a diary, number of completed exercises, number of messages to a coach, number of participants who posted to a discussion board, number of participants who accessed a discussion board (without necessarily posting something), number of visits to the discussion board, number of participants who shared diary entries with other participants, average percentage of pages read, and average percentage of screens viewed. The use of this form was piloted and revised in a group meeting (IB, PM, and BV) on a subset of the included articles (N=150). The resulting data extraction form was then used to extract data from the remaining studies (IB, FB, PM, BV, and AZ). Disagreements regarding the coding were discussed between IB and BV until a consensus was reached.

### Data Analysis

Data were entered into an Excel spreadsheet, which was then converted into an SPSS (Statistical Package for the Social Sciences) file. Each publication was treated as a separate case. Descriptive analyses were performed using SPSS [computer software] (Version 24.0. IBM Corp, Armonk, NY). Absolute and relative frequencies were used as the primary measures for the adherence reporting and defined usage metrics. We tested differences in adherence reporting and usage metrics between studies published in specialized eHealth journals versus nonspecialized journals. Two-sided Chi-squared tests were used, and *P* values<.05 were considered to indicate significant differences between the two groups.

## Results

**Figure 1:**
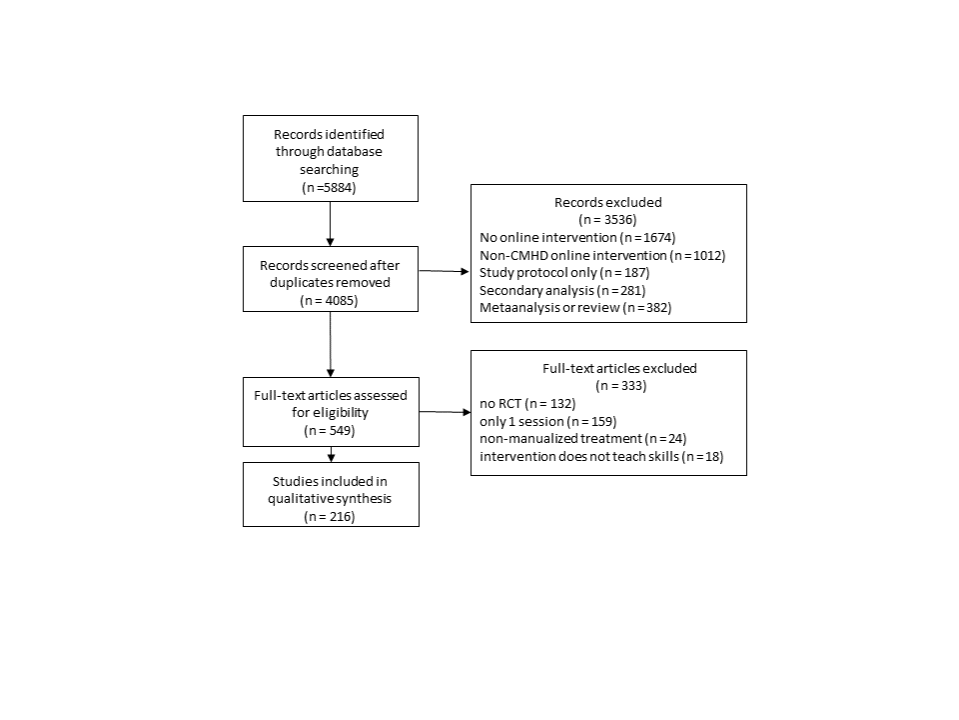
Preferred Reporting Items for Systematic Reviews and Meta-Analyses flow diagram. CMHD: common mental health disorders; RCT: randomized controlled trial.

We identified 216 publications reporting on the primary outcomes of RCTs investigating online interventions for common mental disorders (Figure 1, Multimedia Appendix 1). Of these, 34 were published in specialized eHealth journals (eg, Journal of Medical Internet Research, Internet Interventions, and Computers in Human Behavior) and 182 were published in nonspecialized journals.

Interventions for depression (n=73) were evaluated most frequently, followed by interventions for anxiety disorders (n=65). Substance use disorders (n=27) and eating disorders (n=24) were targeted less frequently. Transdiagnostic interventions were investigated in 27 trials. The majority of trials evaluated interventions aiming to treat mental disorders (n=177); prevention (n=34) and aftercare (n=5) interventions were less frequently investigated.

Table 1 provides details on how adherence was reported in the overall sample of publications as well as how publications from specialized eHealth journals differed from publications in nonspecialized journals. Adherence was not reported at all in 28 publications (13%). The majority of publications (83.3%) included information on adherence in the results section, while less than half (41.2%) included information in the CONSORT statement. Adherence was addressed in the discussion in most publications (69.4%). Approximately one in three publications (30.1%) included information on adherence in the abstract.

Although roughly one in two publications in specialized eHealth journals included information on how adherence was related to outcomes, this was only true for one in four publications in nonspecialized journals (χ^2^_1_=4.6, *P*=.30). In 25 publications (11.6%), correlation or regression analyses were used to investigate the relationship between program usage and outcomes. In 23 publications (10.6%), results of a per protocol analysis that included only participants who completed a preset minimum of the intervention were reported. In eleven publications (5.1%), comparisons between treatment completers and noncompleters or high and low adherers were reported.

In the 188 publications that contained information on adherence, a median of three usage metrics was reported. Of the total of 216 publications, 23 (10.6%) included one metric, 46 (21.3%) included two metrics, 56 (25.9%) included three metrics, and 63 (29.2%) included four or more metrics. Most metrics were related to session/module progression, while the usage of additional intervention features (eg, diaries, discussion boards, and messaging) was rarely reported. The most frequently reported metric was “full intervention completion” (Table 2).

**Table 1:** Adherence reporting.

Adherence reporting		Overall (N=216), n (%)	Studies published in eHealth^a^ journals (n=34), n (%)	Studies published in nonspecialized journals (n=182), n (%)	*P* value
Adherence not addressed at all		28 (13.0)	5 (14.7)	23 (12.6)	.74
Adherence addressed in the abstract		65 (30.1)	12 (35.3)	53 (29.1)	.47
**Adherence addressed in the results section**		180 (83.3)	29 (85.3)	151 (83.0)	.74
	Under own heading	144 (66.7)	24 (70.6)	120 (65.9)	.60
	Adherence related to outcome	62 (28.7)	15 (44.1)	47 (25.8)	.03
Adherence addressed in CONSORT^b^ chart		89 (41.2)	11 (32.4)	78 (42.9)	.22
Adherence addressed in discussion		150 (69.4)	24 (70.6)	126 (69.2)	.88

^a^eHealth: electronic health.

^b^CONSORT: Consolidated Standards of Reporting Trials.

**Table 2:** Usage metrics (used in at least 10 publications).

Usage metrics	Overall (N=216), n (%)	Studies published in eHealth^a^ journals (n=34), n (%)	Studies published in nonspecialized journals (n=182), n (%)	*P* value
Full intervention completion	127 (58.8)	19 (55.9)	108 (59.3)	.71
Completion of set minimum of sessions/modules	99 (45.8)	21 (61.8)	78 (42.9)	.04
Average number of completed sessions/modules	82 (38.0)	9 (26.5)	73 (40.1)	.13
Specified point of intervention dropout (last opened/completed session/module)	71 (32.9)	12 (35.3)	59 (32.4)	.74
Intervention dropout (point not specified)	28 (13.0)	3 (8.8)	5 (2.7)	.43
Number of participants who were allocated to the intervention, but never logged in	68 (31.5)	13 (38.2)	55 (30.2)	.36
Number of times participants were logged on	18 (8.3)	2 (5.8)	16 (8.8)	.57
Total time spent on program	21 (9.7)	0 (0)	21 (11.5)	.04
Number of entries in a diary	12 (5.6)	3 (8.8)	9 (4.9)	.37
Number of messages to a coach	17 (7.9)	0 (0)	17 (9.3)	.06

^a^eHealth: electronic health.

## Discussion

### Principal Findings

The aim of this systematic review was to analyze reporting of adherence to online interventions for common mental disorders and to propose recommendations for reporting adherence in future publications. The majority of publications included information on program usage, but in 13% of the publications, adherence was not referred to at all. Adherence was typically addressed in the results section (often under its own heading), less often in the discussion section, and upfront in the abstract in only in one-third of the publications.

In the majority of publications, multiple usage metrics were reported, which is in line with recommendations given in previous reviews on adherence [9]. Most authors reported usage metrics related to session/module progression, with full intervention completion being the usage metric reported most commonly, while use of intervention components (eg, diaries and discussion boards) was reported less often. Our results are similar to those found in a previous review on predictors of adherence by Donkin and colleagues [14], where module completion was, after the number of logins, the usage metric most commonly reported, whereas usage metrics related to specific intervention components were reported less often. In general, we found considerable variability in the type and number of reported usage metrics. Some of the metrics were only used by specific research groups; for example, the number of pages opened/viewed was only used in seven publications on different trials investigating the eating disorder prevention program “StudentBodies” [34].

Some usage metrics might be more useful or valid than others in terms of how they reflect actual usage behavior. For example, measuring time spent in the intervention may not be the most appropriate metric, as it is still not trivial to determine whether recordings are related to actual intervention use or some other activity in another tab of the same browser or even outside the browser. Completion of exercises throughout the online intervention, on the other hand, might be a user metric that can capture deeper content-focused engagement with the intervention.

In our review, only one in four publications addressed adherence in relation to outcome; publications from specialized eHealth journals did so significantly more often than publications from nonspecialized journals. A previous review [14] reported a slightly higher rate (48%) of studies investigating the impact of adherence to outcome. The authors also investigated the impact of usage metrics on the association between adherence and treatment outcome and suggested that the number of logins and completed modules were associated with effectiveness. In our review, we did not aim to evaluate the relationship between adherence and outcome. However, as findings on the adherence-outcome relationship are inconsistent, it is crucial to conduct such analyses in addition to the primary analysis in future studies on eHealth interventions.

### Proposal for Reporting Standards

Based on our review and previous reviews, we propose reporting standards regarding adherence and usage metrics (Textbox 1). Most importantly, adherence should be addressed in every publication regarding online interventions (ie, in the main outcome paper). Interventions may include different intervention components, but many have similarities regarding their design, such as multiple (consecutive) sessions or modules. Therefore, while some usage metrics are specific to components (eg, use of a diary), others are universal (eg, average number of completed sessions/modules) and should be reported for every intervention. Utilization and reporting of the same usage metrics across interventions facilitates comparison between interventions and studies and allows pooling of data from multiple studies. Hence, it seems reasonable to include usage metrics that have been used most often in the past (ie, information on completed sessions/modules) and to complement them with additional metrics that are appropriate for the intervention based on its design and goals. It is key to include detailed information on how adherence was operationalized and how usage metrics were obtained (eg, how “full intervention completion” was defined). Information on adherence should be included in the abstract, the results, and the CONSORT flow chart. Detailed information on user retention should be included in the results section (eg, in a line chart) to illustrate the rate of use of the intervention by participants according to the module/session. Dichotomization of usage metrics (eg, intervention completion vs noncompletion) should be avoided in favor of continuous measures.

The assessment and report of multiple usage metrics is encouraged, as it has several advantages. First, the use of individual components can be investigated; thus, multiple usage patterns can be identified and ultimately be linked to outcome [12,14]. Second, it offers the possibility to create a composite score consisting of multiple adherence measures that might reflect more facets of adherence, in general. However, it is essential to explain how such composite scores were built and what its single components are. Donkin et al [14] suggested a composite measure including time spent online, completion of activities, and other measures related to an active engagement with the program to be a suitable measure of adherence. Third, reporting of different usage metrics facilitates comparison between interventions and studies on multiple dimensions.

If an intervention includes components like diaries, discussion boards, or messaging tools that are considered an essential part of the intervention by the developers, information on the usage of these components should be provided to allow examination of the actual benefit of the component. Participants in interventions with multiple components may exhibit different usage patterns, and adherence measures should reflect this by including use metrics related to those different components. Analyses of multiple usage metrics can extend our knowledge on the most relevant measures (ie, those closest related to outcome) or parameters a composite score for “overall adherence” should contain.

If possible, adherence should be addressed from two perspectives: progress through the intervention and the level of active engagement with the intervention content. This may help distinguish between users who only “consume” the content (eg, read texts and watch videos) and comply with the protocol and those who actively engage with the intervention (eg, write messages, use diaries, and implement behavior chances). It is, however, a challenge to measure this active engagement, which should therefore be a priority for future research to investigate.

The possible impact of adherence on intervention outcomes should be addressed in the discussion. As appropriate, secondary analyses investigating this impact should be undertaken.

**Standards for reporting adherence.**

- Address adherence in every publication regarding online interventions.
- Provide details on how adherence was operationalized and how usage metrics were obtained in the methods section.
- Include information on adherence in your abstract.
- Provide detailed information on adherence in the results section and the Consolidated Standards of Reporting Trials flow chart:
  - Include detailed information on user retention per session or module. Avoid only reporting dichotomized data (eg, treatment completers vs noncompleters, low vs high adherers).
  - Include at least the following metrics: average number of completed modules/session and number of participants who were allocated to the intervention but never logged in.
  - In interventions with multiple components, include metrics that reflect those different components.
  - Differentiate between passive components (eg, reading assignments and videos) and active components that require engagement (eg, diaries and discussion board). If possible, report adherence to exercises in daily life between intervention sessions.
  - If appropriate, analyze how adherence is related to outcome.
- Address the possible impact of adherence on intervention outcomes in the discussion section.

### Strengths and Limitations

Strengths of this review are the systematic approach and application of independent coding by at least two of the authors, which was employed in every step of the review after initial title and abstract screening. This reduced the risk of selection bias. Our selection procedure led to the inclusion of studies on interventions that are comparable in core design characteristics (eg, multiple sequential sessions). In addition, a large number of publications could be included, reflecting the growth of the field in the past decade.

This review also has some limitations. Initial title and abstract screening was conducted by only one person. Although only publications that were clearly out of the scope of our review were excluded at this step, we cannot completely rule out the possibility that publications were excluded erroneously. Our review is further limited to studies evaluating interventions targeting common mental disorders (depression, anxiety, eating disorders, and substance-related disorders). Interventions targeting other disorders (eg, psychosis or bipolar disorders) were excluded because these conditions are not viewed as common mental disorders. Hence, our findings cannot be generalized across the whole field of electronic mental health research. Moreover, this review cannot draw any conclusions regarding the impact of adherence on outcome (eg, whether there are consistent findings in terms of strength or direction of associations), but examined whether a dose-response relationship was addressed at all in the individual publications. Secondary publications on adherence were not considered, as the CONSORT-HEALTH guidelines (Eysenbach, 2011 [16]) explicitly suggest having information on adherence in main outcome papers.

### Conclusions

In summary, most publications included information on adherence and addressed adherence in the discussion. The most frequently reported usage metric was full intervention completion. There was substantial variety in the usage metrics utilized to operationalize adherence, which impedes comparisons regarding adherence between studies and interventions. Only one in three publications reported on adherence in the abstract. Publications often are screened by abstract and sometimes even evaluated only by the abstract. Results presented in the abstract are more likely to be disseminated by journalists than results presented elsewhere in a manuscript [35]. Hence, to prevent misinterpretation of study results, the abstract should tell the full story, including information on how an intervention was used and how this may have impacted outcomes.

## Appendix

Multimedia Appendix 1 List of included studies.

## Abbreviations

eHealth: electronic health
CONSORT-EHEALTH: Consolidated Standards of Reporting Trials of Electronic and Mobile HEalth Applications and onLine TeleHealth
RCT: randomized controlled trial
