# Supplementary material for: Adherence Reporting in Randomized Controlled Trials Examining Manualized Multisession Online Interventions: Systematic Review of Practices and Proposal for Reporting Standards
Source: J Med Internet Res. 2019 Aug 15;21(8):e14181. doi: 10.2196/14181 (PMC6713038; doi:10.2196/14181)
Supplement: Multimedia Appendix 1 [file jmir_v21i8e14181_app1.pdf]

List of included studies:

	First Author	Year	Journal	Level of care	Disorder group
1	Allen	2016	Bjpsych Open	treatment	anxiety
2	An	2013	Journal of the National Cancer Institute.Monographs	self help	Substance use
3	An	2008	Preventive medicine	self help	substance use
4	Andersson	2017	Behav Ther	treatment	anxiety
5	Andersson	2012	PloS one	treatment	anxiety
6	Andersson	2013	Journal of affective disorders	treatment	depression
7	Andersson	2012	Psychotherapy and psychosomatics	self help	anxiety
8	Andersson	2009	Cognitive behaviour therapy	self help	anxiety
9	Andersson	2013	Cognitive behaviour therapy	self help	anxiety
10	Andrews	2011	Australian and New Zealand Journal of Psychiatry	treatment	anxiety
11	Arean	2016	J Med Internet Res	treatment	depression
12	Beevers	2017	J Consult Clin Psychol	treatment	depression
13	Beiwinkel	2017	J Med Internet Res	treatment	depression
14	Bell	2012	Australian and New Zealand Journal of Psychiatry	treatment	anxiety
15	Berger	2014	Psychotherapy	self help	anxiety
16	Berger	2011	Behaviour research and therapy	self help	anxiety
17	Berger	2011	Cognitive behaviour therapy	self help	depression
18	Berger	2009	Journal of clinical psychology	self help	anxiety
19	Berger	2017	Psychol Med	self help	anxiety
20	Bergstrom	2010	BMC psychiatry	treatment	anxiety
21	Blom	2015	Sleep	treatment	depression
22	Boettcher	2014	Behavior therapy	treatment	anxiety
23	Boettcher	2014	Cognitive behaviour therapy	treatment	anxiety
24	Boss	2018	Addiction	Prevention	substance use
25	Botella	2016	Neuropsychiatric Disease and Treatment	self help	depression
26	Branstrom	2010	American Journal of Health Behavior	self help	substance use
27	Brendryen	2008	Journal of Medical Internet Research	self help	substance use
28	Brendryen	2017	Int J Behav Med	self help	substance use
29	Brendryen	2014	Addiction	self help	substance use
30	Bricker	2013	Nicotine & Tobacco Research	self help	substance use
31	Budney	2015	Psychology of Addictive Behaviors	treatment	substance use
32	Buntrock	2015	Psychotherapy and psychosomatics	self help	depression
33	Buntrock	2016	Jama	Prevention	depression
34	Calear	2016	J Affect Disord	Prevention	anxiety
35	Calear	2009	Journal of consulting and clinical psychology	Prevention	transdiagnostic
36	Canale	2015	American Journal of Drug and Alcohol Abuse	Prevention	anxiety
37	Carlbring	2006	American Journal of Psychiatry	self help	anxiety
38	Carlbring	2007	British Journal of Psychiatry	treatment	anxiety
39	Carlbring	2013	Journal of affective disorders	self help	depression
40	Carrard	2011	Behaviour research and therapy	self help	eating disorder

	First Author	Year	Journal	Level of care	Disorder group
41	Cartreine	2012	Journal of Medical Internet Research	self help	depression
42	Chithambo	2017	Int J Eat Disord	Prevention	eating disorder
43	Choi	2012	Journal of affective disorders	treatment	depression
44	Christensen	2014	Journal of Medical Internet Research	Prevention	anxiety
45	Christensen	2013	BMJ open	treatment	depression
46	Christensen	2006	Psychological medicine	treatment	depression
47	Christoforou	2017	J Med Internet Res	treatment	anxiety
48	Ciuca	2018	J Anxiety Disord	treatment	anxiety
49	Cunningham	2017	Int J Behav Med	self help	substance use
50	Dagoo	2014	Journal of anxiety disorders	self help	anxiety
51	Dahlin	2015	Behaviour research and therapy	treatment	anxiety
52	Day	2013	Behaviour research and therapy	self help	transdiagnostic
53	de Graaf	2009	British Journal of Psychiatry	treatment	depression
54	de Zwaan	2017	Jama Psychiatry	self help	eating disorder
55	Deady	2016	J Med Internet Res	treatment	transdiagnostic
56	Dear	2018	Aust N Z J Psychiatry	treatment	transdiagnostic
57	Dear	2016	J Anxiety Disord	treatment	anxiety
58	Dear	2015	Journal of anxiety disorders	treatment	anxiety
59	Dear	2015	Behavior therapy	treatment	anxiety
60	Donker	2013	Journal of Medical Internet Research	self help	depression
61	Doyle	2008	Journal of Adolescent Health	Prevention	eating disorder
62	Ebert	2013	Psychotherapy and psychosomatics	self help	depression
63	Ebert	2018	Behav Ther	Prevention	depression
64	Farrer	2011	PloS one	self help	depression
65	Fichter	2012	Behaviour research and therapy	aftercare	eating disorder
66	Fogliati	2016	J Anxiety Disord	treatment	anxiety
67	Forand	2018	Behav Ther	treatment	depression
68	Franko	2013	Body Image	Prevention	eating disorder
69	Fucito	2017	Alcohol Clin Exp Res	treatment	substance use
70	Furmark	2009	British Journal of Psychiatry	self help	anxiety
71	Geraedts	2014	Journal of Medical Internet Research	self help	depression
72	Gershkovich	2017	Behav Modif	treatment	anxiety
73	Gilbody	2017	British Journal of Psychiatry	treatment	depression
74	Gilbody	2015	Bmj-British Medical Journal	self help	depression
75	Griffiths	2012	PloS one	self help	depression
76	Hadjistavropoulos	2017	J Anxiety Disord	treatment	transdiagnostic
77	Hallgren	2016	Br J Psychiatry	treatment	depression
78	Hallgren	2015	British Journal of Psychiatry	treatment	depression
79	Hamilton	2017	Pilot Feasibility Stud	Prevention	substance use
80	Hedman	2011	British Journal of Psychiatry	treatment	anxiety
81	Hedman	2011	PloS one	treatment	anxiety
82	Hedman	2014	British Journal of Psychiatry	treatment	anxiety
83	Hickie	2010	Medical Journal of Australia	treatment	depression
84	Hoek	2012	PloS one	self help	transdiagnostic
85	Hollandare	2011	Acta Psychiatrica Scandinavica	aftercare	depression

	First Author	Year	Journal	Level of care	Disorder group
86	Hötzel	2014	Psychological medicine	treatment	eating disorder
87	Imamura	2014	PloS one	self help	depression
88	Ip	2016	Depression and Anxiety	Prevention	depression
89	Ivanova	2016	J Anxiety Disord	treatment	anxiety
90	Jacobi	2017	J Med Internet Res	aftercare	eating disorder
91	Jacobi	2007	International Journal of Eating Disorders	Prevention	eating disorder
92	Jacobi	2012	Behaviour research and therapy	Prevention	eating disorder
93	Jander	2016	J Med Internet Res	Prevention	substance use
94	Johansson	2012	PloS one	self help	depression
95	Johansson	2017	Psychotherapy (Chic)	treatment	anxiety
96	Johansson	2012	PloS one	self help	depression
97	Johnston	2011	PloS one	treatment	anxiety
98	Jonas	2017	Anxiety Stress Coping	self help	depression
99	Jones	2015	Journal of dual diagnosis	Prevention	depression
100	Jones	2008	Pediatrics	Prevention	eating disorder
101	Jones	2016	Journal of anxiety disorders	treatment	anxiety
102	Kass	2014	Behaviour research and therapy	Prevention	eating disorder
103	Kay-Lambkin	2009	Addiction	treatment	substance use
104	Kelders	2015	Behaviour research and therapy	Prevention	depression
105	Kenter	2016	Journal of Medical Internet Research	treatment	depression
106	Khalil	2017	J Med Internet Res	Prevention	substance use
107	Kiropoulos	2008	Journal of anxiety disorders	self help	anxiety
108	Kivi	2014	Cognitive behaviour therapy	treatment	depression
109	Kleiboer	2015	Behaviour research and therapy	treatment	transdiagnostic
110	Klein	2006	Journal of Behavior Therapy and Experimental Psychiatry	treatment	anxiety
111	Klein	2016	Psychother Psychosom	treatment	depression
112	Kok	2015	Psychotherapy and psychosomatics	aftercare	depression
113	Kok	2014	Journal of Medical Internet Research	self help	anxiety
114	Kruger	2017	International Journal of Mental Health Promotion	Prevention	depression
115	Lappalainen	2014	Behaviour research and therapy	self help	depression
116	Lappalainen	2015	Behavior modification	self help	depression
117	Levin	2017	Behavior Modification	Prevention	transdiagnostic
118	Levin	2014	Journal of American College Health	Prevention	transdiagnostic
119	Lintvedt	2013	Clinical psychology & psychotherapy	self help	depression
120	Low	2006	Eating disorders	Prevention	eating disorder
121	Mananes	2014	Journal of Medical Internet Research	self help	substance use
122	McCall	2018	J Med Internet Res	Prevention	anxiety
123	Meyer	2009	Journal of Medical Internet Research	self help	depression
124	Mira	2017	Neuropsychiatr Dis Treat	Prevention	depression
125	Mogoase	2013	Cognitive Therapy and Research	treatment	depression
126	Mohr	2013	PloS one	treatment	depression
127	Montero-Marin	2016	J Med Internet Res	treatment	depression
128	Moritz	2012	Behaviour research and therapy	self help	depression
129	Morris	2015	Anxiety, Stress, and Coping	self help	anxiety

	First Author	Year	Journal	Level of care	Disorder group
130	Munoz	2009	Nicotine & Tobacco Research	self help	substance use
131	Musiat	2014	PloS one	Prevention	transdiagnostic
132	Newby	2013	Psychological medicine	treatment	transdiagnostic
133	Nystrom	2017	J Affect Disord	treatment	depression
134	Oromendia	2016	Cogn Behav Ther	treatment	anxiety
135	Paxling	2011	Cognitive behaviour therapy	treatment	anxiety
136	Perini	2009	Australian and New Zealand Journal of Psychiatry	treatment	depression
137	Phillips	2014	Psychological medicine	self help	depression
138	Pisinger	2010	Journal of Public Health	self help	substance use
139	Postel	2010	Journal of Medical Internet Research	self help	substance use
140	Pots	2016	The British journal of psychiatry : the journal of mental science	self help	depression
141	Powell	2013	Journal of Medical Internet Research	Prevention	transdiagnostic
142	Radhu	2012	Journal of American College Health	self help	transdiagnostic
143	Richards	2015	Behaviour research and therapy	self help	depression
144	Richards	2006	Clinical Psychologist	treatment	anxiety
145	Rickhi	2015	BMC complementary and alternative medicine	self help	depression
146	Riper	2008	Addiction	self help	substance use
147	Robinson	2010	PloS one	self help	anxiety
148	Rollman	2018	Jama Psychiatry	treatment	transdiagnostic
149	Rooke	2013	Journal of Medical Internet Research	self help	substance use
150	Rosmarin	2010	Journal of anxiety disorders	self help	anxiety
151	Rosso	2017	Depression and Anxiety	treatment	depression
152	Ruwaard	2010	Journal of anxiety disorders	self help	anxiety
153	Ruwaard	2013	Clinical psychology & psychotherapy	self help	eating disorder
154	Ruwaard	2009	Cognitive behaviour therapy	treatment	depression
155	Saekow	2015	Internet Interventions	treatment	eating disorder
156	Sanchez	2017	Games Health J	Prevention	transdiagnostic
157	Sanchez-Ortiz	2011	Psychological medicine	treatment	eating disorder
158	Schaub	2012	Journal of Medical Internet Research	self help	substance use
159	Schaub	2015	Journal of Medical Internet Research	self help	substance use
160	Schroder	2017	J Behav Ther Exp Psychiatry	treatment	anxiety
161	Schueller	2013	Clinical psychological science : a journal of the Association for Psychological Science	self help	transdiagnostic
162	Schulz	2016	Behav Res Ther	treatment	anxiety
163	Seidman	2010	Annals of Behavioral Medicine	self help	substance use
164	Sethi	2013	Australian Psychologist	self help	transdiagnostic
165	Sethi	2010	Journal of Technology in Human Services	self help	transdiagnostic
166	Sheeber	2017	J Consult Clin Psychol	treatment	depression
167	Sheeber	2012	Journal of consulting and clinical psychology	treatment	depression
168	Silfvernagel	2012	Journal of Medical Internet Research	treatment	anxiety
169	Spek	2007	Psychological medicine	self help	depression

	First Author	Year	Journal	Level of care	Disorder group
170	Stice	2012	Journal of consulting and clinical psychology	Prevention	eating disorder
171	Stice	2017	J Consult Clin Psychol	Prevention	eating disorder
172	Stolz	2018	J Consult Clin Psychol	treatment	anxiety
173	Strandskov	2017	Behav Ther	treatment	eating disorder
174	Strom	2013	PeerJ	self help	depression
175	Sundstrom	2016	Plos One	treatment	substance use
176	Tait	2014	JMIR mental health	self help	substance use
177	Taylor	2006	Archives of General Psychiatry	Prevention	eating disorder
178	Taylor	2016	Journal of consulting and clinical psychology	Prevention	eating disorder
179	ter Huurne	2015	Journal of Medical Internet Research	treatment	eating disorder
180	Terides	2018	Cogn Behav Ther	treatment	transdiagnostic
181	Tiburcio	2018	Subst Use Misuse	treatment	transdiagnostic
182	Tillfors	2011	Cognitive behaviour therapy	self help	anxiety
183	Tillfors	2008	Depression and anxiety	self help	anxiety
184	Titov	2009	Australian and New Zealand Journal of Psychiatry	treatment	anxiety
185	Titov	2008	Australian and New Zealand Journal of Psychiatry	treatment	anxiety
186	Titov	2010	PloS one	treatment	depression
187	Titov	2010	Behaviour research and therapy	treatment	anxiety
188	Titov	2009	Australian and New Zealand Journal of Psychiatry	treatment	anxiety
189	Titov	2008	Australian and New Zealand Journal of Psychiatry	treatment	anxiety
190	Titov	2008	Australian and New Zealand Journal of Psychiatry	treatment	anxiety
191	Titov	2010	Australian and New Zealand Journal of Psychiatry	treatment	anxiety
192	Titov	2009	Australian and New Zealand Journal of Psychiatry	treatment	anxiety
193	Titov	2015	Behavior therapy	treatment	depression
194	Titov	2013	PloS one	treatment	transdiagnostic
195	Titov	2011	Behaviour research and therapy	treatment	transdiagnostic
196	Titov	2015	Journal of anxiety disorders	treatment	transdiagnostic
197	Titov	2016	Bjpsych Open	treatment	transdiagnostic
198	Tulbure	2015	PloS one	treatment	anxiety
199	Twomey	2014	British Journal of Clinical Psychology	self help	transdiagnostic
200	Unlu Ince	2013	Journal of Medical Internet Research	treatment	depression
201	van Ballegooijen	2013	Journal of Medical Internet Research	self help	anxiety
202	van der Zweerde	2018	Psychol Med	self help	depression
203	van Straten	2008	Journal of Medical Internet Research	self help	transdiagnostic
204	Vernmark	2010	Behaviour research and therapy	self help	depression
205	Wagner	2014	Journal of affective disorders	treatment	depression
206	Wagner	2013	British Journal of Psychiatry	self help	eating disorder
207	Warmerdam	2008	Journal of Medical Internet Research	treatment	depression

	First Author	Year	Journal	Level of care	Disorder group
208	Watts	2013	BMC psychiatry	treatment	depression
209	White	2017	J Health Psychol	treatment	substance use
210	Wilksch	2017	Psychol Med	Prevention	eating disorder
211	Williams	2013	Journal of consulting and clinical psychology	treatment	depression
212	Williams	2015	Journal of affective disorders	treatment	depression
213	Wims	2010	Australian and New Zealand Journal of Psychiatry	treatment	anxiety
214	Yeung	2017	Asian J Psychiatr	treatment	depression
215	Zagorscak	2018	Psychother Psychosom	self help	depression
216	Zwerenz	2017	JMIR Ment Health	aftercare	transdiagnostic

[1-216]

1. Allen AR, Newby JM, Mackenzie A, Smith J, Boulton M, Loughnan SA, et al. Internet cognitive-behavioural treatment for panic disorder: randomised controlled trial and evidence of effectiveness in primary care. BJPsych open. 2016 Mar;2(2):154-62. PMID: 27703768. doi: 10.1192/bjpo.bp.115.001826.
2. An LC, Demers MR, Kirch MA, Considine-Dunn S, Nair V, Dasgupta K, et al. A randomized trial of an avatar-hosted multiple behavior change intervention for young adult smokers. Journal of the National Cancer Institute Monographs. 2013 Dec;2013(47):209-15. PMID: 24395994. doi: 10.1093/jncimonographs/lgt021.
3. An LC, Klatt C, Perry CL, Lein EB, Hennrikus DJ, Pallonen UE, et al. The RealU online cessation intervention for college smokers: a randomized controlled trial. Preventive medicine. 2008 Aug;47(2):194-9. PMID: 18565577. doi: 10.1016/j.ypmed.2008.04.011.
4. Andersson E, Hedman E, Wadstrom O, Boberg J, Andersson EY, Axelsson E, et al. Internet-Based Extinction Therapy for Worry: A Randomized Controlled Trial. Behavior therapy. 2017 May;48(3):391-402. PMID: 28390501. doi: 10.1016/j.beth.2016.07.003.
5. Andersson G, Carlbring P, Furmark T, Group SOFIER. Therapist experience and knowledge acquisition in internet-delivered CBT for social anxiety disorder: a randomized controlled trial. PloS one. 2012;7(5):e37411. PMID: 22649526. doi: 10.1371/journal.pone.0037411.
6. Andersson G, Hesser H, Veilord A, Svedling L, Andersson F, Sleman O, et al. Randomised controlled non-inferiority trial with 3-year follow-up of internet-delivered versus face-to-face group cognitive behavioural therapy for depression. Journal of affective disorders. 2013 Dec;151(3):986-94. PMID: 24035673. doi: 10.1016/j.jad.2013.08.022.
7. Andersson G, Paxling B, Roch-Norlund P, Ostman G, Norgren A, Almlov J, et al. Internet-based psychodynamic versus cognitive behavioral guided self-help for generalized anxiety disorder: a randomized controlled trial. Psychotherapy and psychosomatics. 2012;81(6):344-55. PMID: 22964540. doi: 10.1159/000339371.
8. Andersson G, Waara J, Jonsson U, Malmaeus F, Carlbring P, Ost LG. Internet-based self-help versus one-session exposure in the treatment of spider phobia: a randomized controlled trial. Cognitive behaviour therapy. 2009;38(2):114-20. PMID: 20183690. doi: 10.1080/16506070902931326.
9. Andersson G, Waara J, Jonsson U, Malmaeus F, Carlbring P, Ost LG. Internet-based exposure treatment versus one-session exposure treatment of snake phobia: a randomized controlled trial. Cognitive behaviour therapy. 2013;42(4):284-91. PMID: 24245707. doi: 10.1080/16506073.2013.844202.

10. Andrews G, Davies M, Titov N. Effectiveness randomized controlled trial of face to face versus Internet cognitive behaviour therapy for social phobia. The Australian and New Zealand journal of psychiatry. 2011 Apr;45(4):337-40. PMID: 21323490. doi: 10.3109/00048674.2010.538840.
11. Arean PA, Hallgren KA, Jordan JT, Gazzaley A, Atkins DC, Heagerty PJ, et al. The Use and Effectiveness of Mobile Apps for Depression: Results From a Fully Remote Clinical Trial. Journal of medical Internet research. 2016 Dec 20;18(12):e330. PMID: 27998876. doi: 10.2196/jmir.6482.
12. Beevers CG, Pearson R, Hoffman JS, Foulser AA, Shumake J, Meyer B. Effectiveness of an internet intervention (Deprexis) for depression in a united states adult sample: A parallel-group pragmatic randomized controlled trial. Journal of consulting and clinical psychology. 2017 Apr;85(4):367-80. PMID: 28230390. doi: 10.1037/ccp0000171.
13. Beiwinkel T, Eissing T, Telle NT, Siegmund-Schultze E, Rossler W. Effectiveness of a Web-Based Intervention in Reducing Depression and Sickness Absence: Randomized Controlled Trial. Journal of medical Internet research. 2017 Jun 15;19(6):e213. PMID: 28619701. doi: 10.2196/jmir.6546.
14. Bell CJ, Colhoun HC, Carter FA, Frampton CM. Effectiveness of computerised cognitive behaviour therapy for anxiety disorders in secondary care. The Australian and New Zealand journal of psychiatry. 2012 Jul;46(7):630-40. PMID: 22327097. doi: 10.1177/0004867412437345.
15. Berger T, Boettcher J, Caspar F. Internet-based guided self-help for several anxiety disorders: a randomized controlled trial comparing a tailored with a standardized disorder-specific approach. Psychotherapy (Chicago, Ill). 2014 Jun;51(2):207-19. PMID: 24041199. doi: 10.1037/a0032527.
16. Berger T, Caspar F, Richardson R, Kneubuhler B, Sutter D, Andersson G. Internet-based treatment of social phobia: a randomized controlled trial comparing unguided with two types of guided self-help. Behaviour research and therapy. 2011 Mar;49(3):158-69. PMID: 21255767. doi: 10.1016/j.brat.2010.12.007.
17. Berger T, Hammerli K, Gubser N, Andersson G, Caspar F. Internet-based treatment of depression: a randomized controlled trial comparing guided with unguided self-help. Cognitive behaviour therapy. 2011;40(4):251-66. PMID: 22060248. doi: 10.1080/16506073.2011.616531.
18. Berger T, Hohl E, Caspar F. Internet-based treatment for social phobia: a randomized controlled trial. Journal of clinical psychology. 2009 Oct;65(10):1021-35. PMID: 19437505. doi: 10.1002/jclp.20603.
19. Berger T, Urech A, Krieger T, Stolz T, Schulz A, Vincent A, et al. Effects of a transdiagnostic unguided Internet intervention ('velibra') for anxiety disorders in primary care: results of a randomized controlled trial. Psychological medicine. 2017 Jan;47(1):67-80. PMID: 27655039. doi: 10.1017/S0033291716002270.
20. Bergstrom J, Andersson G, Ljotsson B, Ruck C, Andreewitch S, Karlsson A, et al. Internet-versus group-administered cognitive behaviour therapy for panic disorder in a psychiatric setting: a randomised trial. BMC psychiatry. 2010 Jul 2;10(Journal Article):54. PMID: 20598127. doi: 10.1186/1471-244X-10-54.
21. Blom K, Jernelov S, Kraepelien M, Bergdahl MO, Jungmarker K, Ankartjarn L, et al. Internet treatment addressing either insomnia or depression, for patients with both diagnoses: a randomized trial. Sleep. 2015 Feb 1;38(2):267-77. PMID: 25337948. doi: 10.5665/sleep.4412.
22. Boettcher J, Astrom V, Pahlsson D, Schenstrom O, Andersson G, Carlbring P. Internet-based mindfulness treatment for anxiety disorders: a randomized controlled trial. Behavior therapy. 2014 Mar;45(2):241-53. PMID: 24491199. doi: 10.1016/j.beth.2013.11.003.
23. Boettcher J, Hasselrot J, Sund E, Andersson G, Carlbring P. Combining attention training with internet-based cognitive-behavioural self-help for social anxiety: a randomised controlled trial. Cognitive behaviour therapy. 2014;43(1):34-48. PMID: 23898817. doi: 10.1080/16506073.2013.809141.
24. Boss L, Lehr D, Schaub MP, Paz Castro R, Riper H, Berking M, et al. Efficacy of a web-based intervention with and without guidance for employees with risky drinking: results of a three-arm randomized controlled trial. Addiction (Abingdon, England). 2018 Apr;113(4):635-46. PMID: 29105879. doi: 10.1111/add.14085.

25. Botella C, Mira A, Moragrega I, Garcia-Palacios A, Breton-Lopez J, Castilla D, et al. An Internet-based program for depression using activity and physiological sensors: efficacy, expectations, satisfaction, and ease of use. Neuropsychiatr Dis Treat. 2016;12:393-406. PMID: 27042067. doi: 10.2147/NDT.S93315.
26. Branstrom R, Penilla C, Perez-Stable EJ, Munoz RF. Positive affect and mood management in successful smoking cessation. American journal of health behavior. 2010 Sep-Oct;34(5):553-62. PMID: 20524885.
27. Brendryen H, Drozd F, Kraft P. A digital smoking cessation program delivered through internet and cell phone without nicotine replacement (happy ending): randomized controlled trial. Journal of medical Internet research. 2008 Nov 28;10(5):e51. PMID: 19087949. doi: 10.2196/jmir.1005.
28. Brendryen H, Johansen A, Duckert F, Nesvag S. A Pilot Randomized Controlled Trial of an Internet-Based Alcohol Intervention in a Workplace Setting. International journal of behavioral medicine. 2017 Oct;24(5):768-77. PMID: 28755326. doi: 10.1007/s12529-017-9665-0.
29. Brendryen H, Lund IO, Johansen AB, Riksheim M, Nesvag S, Duckert F. Balance--a pragmatic randomized controlled trial of an online intensive self-help alcohol intervention. Addiction (Abingdon, England). 2014 Feb;109(2):218-26. PMID: 24134709. doi: 10.1111/add.12383.
30. Bricker J, Wyszynski C, Comstock B, Heffner JL. Pilot randomized controlled trial of web-based acceptance and commitment therapy for smoking cessation. Nicotine & tobacco research : official journal of the Society for Research on Nicotine and Tobacco. 2013 Oct;15(10):1756-64. PMID: 23703730. doi: 10.1093/ntr/ntt056.
31. Budney AJ, Stanger C, Tilford JM, Scherer EB, Brown PC, Li Z, et al. Computer-assisted behavioral therapy and contingency management for cannabis use disorder. Psychology of addictive behaviors : journal of the Society of Psychologists in Addictive Behaviors. 2015 Sep;29(3):501-11. PMID: 25938629. doi: 10.1037/adb0000078.
32. Buntrock C, Ebert D, Lehr D, Riper H, Smit F, Cuijpers P, et al. Effectiveness of a web-based cognitive behavioural intervention for subthreshold depression: pragmatic randomised controlled trial. Psychotherapy and psychosomatics. 2015;84(6):348-58. PMID: 26398885. doi: 10.1159/000438673.
33. Buntrock C, Ebert DD, Lehr D, Smit F, Riper H, Berking M, et al. Effect of a Web-Based Guided Self-help Intervention for Prevention of Major Depression in Adults With Subthreshold Depression: A Randomized Clinical Trial. Jama. 2016 May 3;315(17):1854-63. PMID: 27139058. doi: 10.1001/jama.2016.4326.
34. Calear AL, Batterham PJ, Poyser CT, Mackinnon AJ, Griffiths KM, Christensen H. Cluster randomised controlled trial of the e-couch Anxiety and Worry program in schools. Journal of affective disorders. 2016 May 15;196:210-7. PMID: 26926660. doi: 10.1016/j.jad.2016.02.049.
35. Calear AL, Christensen H, Mackinnon A, Griffiths KM, O'Kearney R. The YouthMood Project: a cluster randomized controlled trial of an online cognitive behavioral program with adolescents. Journal of consulting and clinical psychology. 2009 Dec;77(6):1021-32. PMID: 19968379. doi: 10.1037/a0017391.
36. Canale N, Vieno A, Santinello M, Chieco F, Andriolo S. The efficacy of computerized alcohol intervention tailored to drinking motives among college students: a quasi-experimental pilot study. The American journal of drug and alcohol abuse. 2015 Mar;41(2):183-7. PMID: 25700006. doi: 10.3109/00952990.2014.991022.
37. Carlbring P, Bohman S, Brunt S, Buhrman M, Westling BE, Ekselius L, et al. Remote treatment of panic disorder: a randomized trial of internet-based cognitive behavior therapy supplemented with telephone calls. The American journal of psychiatry. 2006 Dec;163(12):2119-25. PMID: 17151163. doi: 10.1176/ajp.2006.163.12.2119.
38. Carlbring P, Gunnarsdottir M, Hedensjo L, Andersson G, Ekselius L, Furmark T. Treatment of social phobia: randomised trial of internet-delivered cognitive-behavioural therapy with telephone support. The British journal of psychiatry : the journal of mental science. 2007 Feb;190(Journal Article):123-8. PMID: 17267928. doi: 10.1192/bjp.bp.105.020107.

39. Carlbring P, Hagglund M, Luthstrom A, Dahlin M, Kadowaki A, Vernmark K, et al. Internet-based behavioral activation and acceptance-based treatment for depression: a randomized controlled trial. Journal of affective disorders. 2013 Jun;148(2-3):331-7. PMID: 23357657. doi: 10.1016/j.jad.2012.12.020.
40. Carrard I, Crepin C, Rouget P, Lam T, Golay A, Van der Linden M. Randomised controlled trial of a guided self-help treatment on the Internet for binge eating disorder. Behaviour research and therapy. 2011 Aug;49(8):482-91. PMID: 21641580. doi: 10.1016/j.brat.2011.05.004.
41. Cartreine JA, Locke SE, Buckey JC, Sandoval L, Hegel MT. Electronic problem-solving treatment: description and pilot study of an interactive media treatment for depression. JMIR research protocols. 2012 Sep 25;1(2):e11. PMID: 23611902. doi: 10.2196/resprot.1925.
42. Chithambo TP, Huey SJ, Jr. Internet-delivered eating disorder prevention: A randomized controlled trial of dissonance-based and cognitive-behavioral interventions. The International journal of eating disorders. 2017 Oct;50(10):1142-51. PMID: 28801926. doi: 10.1002/eat.22762.
43. Choi I, Zou J, Titov N, Dear BF, Li S, Johnston L, et al. Culturally attuned Internet treatment for depression amongst Chinese Australians: a randomised controlled trial. Journal of affective disorders. 2012 Feb;136(3):459-68. PMID: 22177742. doi: 10.1016/j.jad.2011.11.003.
44. Christensen H, Batterham P, Mackinnon A, Griffiths KM, Kalia Hehir K, Kenardy J, et al. Prevention of generalized anxiety disorder using a web intervention, iChill: randomized controlled trial. Journal of medical Internet research. 2014 Sep 2;16(9):e199. PMID: 25270886. doi: 10.2196/jmir.3507.
45. Christensen H, Farrer L, Batterham PJ, Mackinnon A, Griffiths KM, Donker T. The effect of a web-based depression intervention on suicide ideation: secondary outcome from a randomised controlled trial in a helpline. BMJ open. 2013 Jun 28;3(6). PMID: 23811172. doi: 10.1136/bmjopen-2013-002886.
46. Christensen H, Griffiths KM, Mackinnon AJ, Brittliffe K. Online randomized controlled trial of brief and full cognitive behaviour therapy for depression. Psychological medicine. 2006 Dec;36(12):1737-46. PMID: 16938144. doi: 10.1017/S0033291706008695.
47. Christoforou M, Saez Fonseca JA, Tsakanikos E. Two Novel Cognitive Behavioral Therapy-Based Mobile Apps for Agoraphobia: Randomized Controlled Trial. Journal of medical Internet research. 2017 Nov 24;19(11):e398. PMID: 29175809. doi: 10.2196/jmir.7747.
48. Ciuca AM, Berger T, Crisan LG, Miclea M. Internet-based treatment for panic disorder: A three-arm randomized controlled trial comparing guided (via real-time video sessions) with unguided self-help treatment and a waitlist control. PAXPD study results. Journal of anxiety disorders. 2018 May;56:43-55. PMID: 29625834. doi: 10.1016/j.janxdis.2018.03.009.
49. Cunningham JA, Shorter GW, Murphy M, Kushnir V, Rehm J, Hendershot CS. Randomized Controlled Trial of a Brief Versus Extended Internet Intervention for Problem Drinkers. International journal of behavioral medicine. 2017 Oct;24(5):760-7. PMID: 27770293. doi: 10.1007/s12529-016-9604-5.
50. Dagoo J, Asplund RP, Bsenko HA, Hjerling S, Holmberg A, Westh S, et al. Cognitive behavior therapy versus interpersonal psychotherapy for social anxiety disorder delivered via smartphone and computer: a randomized controlled trial. Journal of anxiety disorders. 2014 May;28(4):410-7. PMID: 24731441. doi: 10.1016/j.janxdis.2014.02.003.
51. Dahlin M, Andersson G, Magnusson K, Johansson T, Sjogren J, Hakansson A, et al. Internet-delivered acceptance-based behaviour therapy for generalized anxiety disorder: A randomized controlled trial. Behaviour research and therapy. 2016 Feb;77(Journal Article):86-95. PMID: 26731173. doi: 10.1016/j.brat.2015.12.007.
52. Day V, McGrath PJ, Wojtowicz M. Internet-based guided self-help for university students with anxiety, depression and stress: a randomized controlled clinical trial. Behaviour research and therapy. 2013 Jul;51(7):344-51. PMID: 23639300. doi: 10.1016/j.brat.2013.03.003.
53. de Graaf LE, Gerhards SA, Arntz A, Riper H, Metsemakers JF, Evers SM, et al. Clinical effectiveness of online computerised cognitive-behavioural therapy without support for depression in primary care: randomised trial. The British journal of psychiatry : the journal of mental science. 2009 Jul;195(1):73-80. PMID: 19567900. doi: 10.1192/bjp.bp.108.054429.

54. de Zwaan M, Herpertz S, Zipfel S, Svaldi J, Friederich HC, Schmidt F, et al. Effect of Internet-Based Guided Self-help vs Individual Face-to-Face Treatment on Full or Subsyndromal Binge Eating Disorder in Overweight or Obese Patients: The INTERBED Randomized Clinical Trial. JAMA psychiatry. 2017 Oct 1;74(10):987-95. PMID: 28768334. doi: 10.1001/jamapsychiatry.2017.2150.
55. Deady M, Mills KL, Teesson M, Kay-Lambkin F. An Online Intervention for Co-Occurring Depression and Problematic Alcohol Use in Young People: Primary Outcomes From a Randomized Controlled Trial. Journal of medical Internet research. 2016 Mar 23;18(3):e71. PMID: 27009465. doi: 10.2196/jmir.5178.
56. Dear BF, Fogliati VJ, Fogliati R, Johnson B, Boyle O, Karin E, et al. Treating anxiety and depression in young adults: A randomised controlled trial comparing clinician-guided versus self-guided Internet-delivered cognitive behavioural therapy. The Australian and New Zealand journal of psychiatry. 2018 Jul;52(7):668-79. PMID: 29064283. doi: 10.1177/0004867417738055.
57. Dear BF, Staples LG, Terides MD, Fogliati VJ, Sheehan J, Johnston L, et al. Transdiagnostic versus disorder-specific and clinician-guided versus self-guided internet-delivered treatment for Social Anxiety Disorder and comorbid disorders: A randomized controlled trial. Journal of anxiety disorders. 2016 Aug;42:30-44. PMID: 27261562. doi: 10.1016/j.janxdis.2016.05.004.
58. Dear BF, Staples LG, Terides MD, Karin E, Zou J, Johnston L, et al. Transdiagnostic versus disorder-specific and clinician-guided versus self-guided internet-delivered treatment for generalized anxiety disorder and comorbid disorders: A randomized controlled trial. Journal of anxiety disorders. 2015 Dec;36(Journal Article):63-77. PMID: 26460536. doi: 10.1016/j.janxdis.2015.09.003.
59. Dear BF, Zou JB, Ali S, Lorian CN, Johnston L, Sheehan J, et al. Clinical and cost-effectiveness of therapist-guided internet-delivered cognitive behavior therapy for older adults with symptoms of anxiety: a randomized controlled trial. Behavior therapy. 2015 Mar;46(2):206-17. PMID: 25645169. doi: 10.1016/j.beth.2014.09.007.
60. Donker T, Bennett K, Bennett A, Mackinnon A, van Straten A, Cuijpers P, et al. Internet-delivered interpersonal psychotherapy versus internet-delivered cognitive behavioral therapy for adults with depressive symptoms: randomized controlled noninferiority trial. Journal of medical Internet research. 2013 May 13;15(5):e82. PMID: 23669884. doi: 10.2196/jmir.2307.
61. Doyle AC, Goldschmidt A, Huang C, Winzelberg AJ, Taylor CB, Wilfley DE. Reduction of overweight and eating disorder symptoms via the Internet in adolescents: a randomized controlled trial. The Journal of adolescent health : official publication of the Society for Adolescent Medicine. 2008 Aug;43(2):172-9. PMID: 18639791. doi: 10.1016/j.jadohealth.2008.01.011.
62. Ebert D, Tarnowski T, Gollwitzer M, Sieland B, Berking M. A transdiagnostic internet-based maintenance treatment enhances the stability of outcome after inpatient cognitive behavioral therapy: a randomized controlled trial. Psychotherapy and psychosomatics. 2013;82(4):246-56. PMID: 23736751. doi: 10.1159/000345967.
63. Ebert DD, Buntrock C, Lehr D, Smit F, Riper H, Baumeister H, et al. Effectiveness of Web- and Mobile-Based Treatment of Subthreshold Depression With Adherence-Focused Guidance: A Single-Blind Randomized Controlled Trial. Behavior therapy. 2018 Jan;49(1):71-83. PMID: 29405923. doi: 10.1016/j.beth.2017.05.004.
64. Farrer L, Christensen H, Griffiths KM, Mackinnon A. Internet-based CBT for depression with and without telephone tracking in a national helpline: randomised controlled trial. PloS one. 2011;6(11):e28099. PMID: 22140514. doi: 10.1371/journal.pone.0028099.
65. Fichter MM, Quadflieg N, Nisslmuller K, Lindner S, Osen B, Huber T, et al. Does internet-based prevention reduce the risk of relapse for anorexia nervosa? Behaviour research and therapy. 2012 Mar;50(3):180-90. PMID: 22317754. doi: 10.1016/j.brat.2011.12.003.
66. Fogliati VJ, Dear BF, Staples LG, Terides MD, Sheehan J, Johnston L, et al. Disorder-specific versus transdiagnostic and clinician-guided versus self-guided internet-delivered treatment for panic disorder and comorbid disorders: A randomized controlled trial. Journal of anxiety disorders. 2016 Apr;39:88-102. PMID: 27003376. doi: 10.1016/j.janxdis.2016.03.005.
67. Forand NR, Barnett JG, Strunk DR, Hindiyeh MU, Feinberg JE, Keefe JR. Efficacy of Guided iCBT for Depression and Mediation of Change by Cognitive Skill Acquisition. Behavior therapy. 2018 Mar;49(2):295-307. PMID: 29530267. doi: 10.1016/j.beth.2017.04.004.

68. Franko DL, Cousineau TM, Rodgers RF, Roehrig JP. BodiMojo: effective Internet-based promotion of positive body image in adolescent girls. Body image. 2013 Sep;10(4):481-8. PMID: 23768797. doi: 10.1016/j.bodyim.2013.04.008.
69. Fucito LM, DeMartini KS, Hanrahan TH, Yaggi HK, Heffern C, Redeker NS. Using Sleep Interventions to Engage and Treat Heavy-Drinking College Students: A Randomized Pilot Study. Alcoholism, clinical and experimental research. 2017 Apr;41(4):798-809. PMID: 28118486. doi: 10.1111/acer.13342.
70. Furmark T, Carlbring P, Hedman E, Sonnenstein A, Clevberger P, Bohman B, et al. Guided and unguided self-help for social anxiety disorder: randomised controlled trial. The British journal of psychiatry : the journal of mental science. 2009 Nov;195(5):440-7. PMID: 19880935. doi: 10.1192/bjp.bp.108.060996.
71. Geraedts AS, Kleiboer AM, Wiezer NM, van Mechelen W, Cuijpers P. Short-term effects of a web-based guided self-help intervention for employees with depressive symptoms: randomized controlled trial. Journal of medical Internet research. 2014 May 6;16(5):e121. PMID: 24800966. doi: 10.2196/jmir.3185.
72. Gershkovich M, Herbert JD, Forman EM, Schumacher LM, Fischer LE. Internet-Delivered Acceptance-Based Cognitive-Behavioral Intervention for Social Anxiety Disorder With and Without Therapist Support: A Randomized Trial. Behavior modification. 2017 Sep;41(5):583-608. PMID: 28776431. doi: 10.1177/0145445517694457.
73. Gilbody S, Brabyn S, Lovell K, Kessler D, Devlin T, Smith L, et al. Telephone-supported computerised cognitive-behavioural therapy: REEACT-2 large-scale pragmatic randomised controlled trial. The British journal of psychiatry : the journal of mental science. 2017 May;210(5):362-7. PMID: 28254959. doi: 10.1192/bjp.bp.116.192435.
74. Gilbody S, Littlewood E, Hewitt C, Brierley G, Tharmanathan P, Araya R, et al. Computerised cognitive behaviour therapy (cCBT) as treatment for depression in primary care (REEACT trial): large scale pragmatic randomised controlled trial. BMJ. 2015 Nov 11;351(Journal Article):h5627. PMID: 26559241. doi: 10.1136/bmj.h5627.
75. Griffiths KM, Mackinnon AJ, Crisp DA, Christensen H, Bennett K, Farrer L. The effectiveness of an online support group for members of the community with depression: a randomised controlled trial. PloS one. 2012;7(12):e53244. PMID: 23285271. doi: 10.1371/journal.pone.0053244.
76. Hadjistavropoulos HD, Schneider LH, Edmonds M, Karin E, Nugent MN, Dirkse D, et al. Randomized controlled trial of internet-delivered cognitive behaviour therapy comparing standard weekly versus optional weekly therapist support. Journal of anxiety disorders. 2017 Dec;52:15-24. PMID: 28964994. doi: 10.1016/j.janxdis.2017.09.006.
77. Hallgren M, Helgadottir B, Herring MP, Zeebari Z, Lindefors N, Kaldo V, et al. Exercise and internet-based cognitive-behavioural therapy for depression: multicentre randomised controlled trial with 12-month follow-up. The British journal of psychiatry : the journal of mental science. 2016 Nov;209(5):414-20. PMID: 27609813. doi: 10.1192/bjp.bp.115.177576.
78. Hallgren M, Kraepelien M, Ojehagen A, Lindefors N, Zeebari Z, Kaldo V, et al. Physical exercise and internet-based cognitive-behavioural therapy in the treatment of depression: randomised controlled trial. The British journal of psychiatry : the journal of mental science. 2015 Sep;207(3):227-34. PMID: 26089305. doi: 10.1192/bjp.bp.114.160101.
79. Hamilton FL, Hornby J, Sheringham J, Linke S, Ashton C, Moore K, et al. DIAMOND (DIgital Alcohol Management ON Demand): a mixed methods feasibility RCT and embedded process evaluation of a digital health intervention to reduce hazardous and harmful alcohol use. Pilot and feasibility studies. 2017;3:34. PMID: 28879021. doi: 10.1186/s40814-017-0177-0.
80. Hedman E, Andersson G, Andersson E, Ljotsson B, Ruck C, Asmundson GJ, et al. Internet-based cognitive-behavioural therapy for severe health anxiety: randomised controlled trial. The British journal of psychiatry : the journal of mental science. 2011 Mar;198(3):230-6. PMID: 21357882. doi: 10.1192/bjp.bp.110.086843.
81. Hedman E, Andersson G, Ljotsson B, Andersson E, Ruck C, Mortberg E, et al. Internet-based cognitive behavior therapy vs. cognitive behavioral group therapy for social anxiety disorder: a

randomized controlled non-inferiority trial. PloS one. 2011 Mar 25;6(3):e18001. PMID: 21483704. doi: 10.1371/journal.pone.0018001.
82. Hedman E, Axelsson E, Gorling A, Ritzman C, Ronnheden M, El Alaoui S, et al. Internet-delivered exposure-based cognitive-behavioural therapy and behavioural stress management for severe health anxiety: randomised controlled trial. The British journal of psychiatry : the journal of mental science. 2014 Oct;205(4):307-14. PMID: 25104835. doi: 10.1192/bjp.bp.113.140913.
83. Hickie IB, Davenport TA, Luscombe GM, Moore M, Griffiths KM, Christensen H. Practitioner-supported delivery of internet-based cognitive behaviour therapy: evaluation of the feasibility of conducting a cluster randomised trial. The Medical journal of Australia. 2010 Jun 7;192(11 Suppl):S31-5. PMID: 20528705.
84. Hoek W, Schuurmans J, Koot HM, Cuijpers P. Effects of Internet-based guided self-help problem-solving therapy for adolescents with depression and anxiety: a randomized controlled trial. PloS one. 2012;7(8):e43485. PMID: 22952691. doi: 10.1371/journal.pone.0043485.
85. Hollandare F, Johnsson S, Randestad M, Tillfors M, Carlbring P, Andersson G, et al. Randomized trial of Internet-based relapse prevention for partially remitted depression. Acta psychiatrica Scandinavica. 2011 Oct;124(4):285-94. PMID: 21401534. doi: 10.1111/j.1600-0447.2011.01698.x.
86. Hotzel K, von Brachel R, Schmidt U, Rieger E, Kosfelder J, Hechler T, et al. An Internet-based program to enhance motivation to change in females with symptoms of an eating disorder: a randomized controlled trial. Psychological medicine. 2014 Jul;44(9):1947-63. PMID: 24128818. doi: 10.1017/S0033291713002481.
87. Imamura K, Kawakami N, Furukawa TA, Matsuyama Y, Shimazu A, Umanodan R, et al. Effects of an Internet-based cognitive behavioral therapy (iCBT) program in Manga format on improving subthreshold depressive symptoms among healthy workers: a randomized controlled trial. PloS one. 2014;9(5):e97167. PMID: 24844530. doi: 10.1371/journal.pone.0097167.
88. Ip P, Chim D, Chan KL, Li TM, Ho FK, Van Voorhees BW, et al. Effectiveness of a culturally attuned Internet-based depression prevention program for Chinese adolescents: A randomized controlled trial. Depress Anxiety. 2016 Dec;33(12):1123-31. PMID: 27618799. doi: 10.1002/da.22554.
89. Ivanova E, Lindner P, Ly KH, Dahlin M, Vernmark K, Andersson G, et al. Guided and unguided Acceptance and Commitment Therapy for social anxiety disorder and/or panic disorder provided via the Internet and a smartphone application: A randomized controlled trial. Journal of anxiety disorders. 2016 Dec;44:27-35. PMID: 27721123. doi: 10.1016/j.janxdis.2016.09.012.
90. Jacobi C, Beintner I, Fittig E, Trockel M, Braks K, Schade-Brittinger C, et al. Web-Based Aftercare for Women With Bulimia Nervosa Following Inpatient Treatment: Randomized Controlled Efficacy Trial. Journal of medical Internet research. 2017 Sep 22;19(9):e321. PMID: 28939544. doi: 10.2196/jmir.7668.
91. Jacobi C, Morris L, Beckers C, Bronisch-Holtze J, Winter J, Winzelberg AJ, et al. Maintenance of internet-based prevention: a randomized controlled trial. The International journal of eating disorders. 2007 Mar;40(2):114-9. PMID: 17080447. doi: 10.1002/eat.20344.
92. Jacobi C, Volker U, Trockel MT, Taylor CB. Effects of an Internet-based intervention for subthreshold eating disorders: a randomized controlled trial. Behaviour research and therapy. 2012 Feb;50(2):93-9. PMID: 22137366. doi: 10.1016/j.brat.2011.09.013.
93. Jander A, Crutzen R, Mercken L, Candel M, de Vries H. Effects of a Web-Based Computer-Tailored Game to Reduce Binge Drinking Among Dutch Adolescents: A Cluster Randomized Controlled Trial. Journal of medical Internet research. 2016 Feb 3;18(2):e29. PMID: 26842694. doi: 10.2196/jmir.4708.
94. Johansson R, Ekbladh S, Hebert A, Lindstrom M, Moller S, Petitt E, et al. Psychodynamic guided self-help for adult depression through the internet: a randomised controlled trial. PloS one. 2012;7(5):e38021. PMID: 22741027. doi: 10.1371/journal.pone.0038021.
95. Johansson R, Hesslow T, Ljotsson B, Jansson A, Jonsson L, Fardig S, et al. Internet-based affect-focused psychodynamic therapy for social anxiety disorder: A randomized controlled trial with 2-year follow-up. Psychotherapy (Chicago, Ill). 2017 Dec;54(4):351-60. PMID: 29251954. doi: 10.1037/pst0000147.

96. Johansson R, Sjoberg E, Sjogren M, Johnsson E, Carlbring P, Andersson T, et al. Tailored vs. standardized internet-based cognitive behavior therapy for depression and comorbid symptoms: a randomized controlled trial. PloS one. 2012;7(5):e36905. PMID: 22615841. doi: 10.1371/journal.pone.0036905.
97. Johnston L, Titov N, Andrews G, Spence J, Dear BF. A RCT of a transdiagnostic internet-delivered treatment for three anxiety disorders: examination of support roles and disorder-specific outcomes. PloS one. 2011;6(11):e28079. PMID: 22132216. doi: 10.1371/journal.pone.0028079.
98. Jonas B, Leuschner F, Tossmann P. Efficacy of an internet-based intervention for burnout: a randomized controlled trial in the German working population. Anxiety, stress, and coping. 2017 Mar;30(2):133-44. PMID: 27602992. doi: 10.1080/10615806.2016.1233324.
99. Jones HA, Heffner JL, Mercer L, Wyszynski CM, Vilardaga R, Bricker JB. Web-based acceptance and commitment therapy smoking cessation treatment for smokers with depressive symptoms. Journal of dual diagnosis. 2015;11(1):56-62. PMID: 25671683. doi: 10.1080/15504263.2014.992588.
100. Jones M, Luce KH, Osborne MI, Taylor K, Cunning D, Doyle AC, et al. Randomized, controlled trial of an internet-facilitated intervention for reducing binge eating and overweight in adolescents. Pediatrics. 2008 Mar;121(3):453-62. PMID: 18310192. doi: 10.1542/peds.2007-1173.
101. Jones SL, Hadjistavropoulos HD, Soucy JN. A randomized controlled trial of guided internet-delivered cognitive behaviour therapy for older adults with generalized anxiety. Journal of anxiety disorders. 2016 Jan;37(Journal Article):1-9. PMID: 26561733. doi: 10.1016/j.janxdis.2015.10.006.
102. Kass AE, Trockel M, Safer DL, Sinton MM, Cunning D, Rizk MT, et al. Internet-based preventive intervention for reducing eating disorder risk: A randomized controlled trial comparing guided with unguided self-help. Behaviour research and therapy. 2014 Dec;63(Journal Article):90-8. PMID: 25461783. doi: 10.1016/j.brat.2014.09.010.
103. Kay-Lambkin FJ, Baker AL, Lewin TJ, Carr VJ. Computer-based psychological treatment for comorbid depression and problematic alcohol and/or cannabis use: a randomized controlled trial of clinical efficacy. Addiction (Abingdon, England). 2009 Mar;104(3):378-88. PMID: 19207345. doi: 10.1111/j.1360-0443.2008.02444.x.
104. Kelders SM, Bohlmeijer ET, Pots WT, van Gemert-Pijnen JE. Comparing human and automated support for depression: Fractional factorial randomized controlled trial. Behaviour research and therapy. 2015 Sep;72(Journal Article):72-80. PMID: 26196078. doi: 10.1016/j.brat.2015.06.014.
105. Kenter RM, Cuijpers P, Beekman A, van Straten A. Effectiveness of a Web-Based Guided Self-help Intervention for Outpatients With a Depressive Disorder: Short-term Results From a Randomized Controlled Trial. Journal of medical Internet research. 2016 Mar 31;18(3):e80. PMID: 27032449. doi: 10.2196/jmir.4861.
106. Khalil GE, Wang H, Calabro KS, Mitra N, Shegog R, Prokhorov AV. From the Experience of Interactivity and Entertainment to Lower Intention to Smoke: A Randomized Controlled Trial and Path Analysis of a Web-Based Smoking Prevention Program for Adolescents. Journal of medical Internet research. 2017 Feb 16;19(2):e44. PMID: 28209560. doi: 10.2196/jmir.7174.
107. Kiropoulos LA, Klein B, Austin DW, Gilson K, Pier C, Mitchell J, et al. Is internet-based CBT for panic disorder and agoraphobia as effective as face-to-face CBT? Journal of anxiety disorders. 2008 Dec;22(8):1273-84. PMID: 18289829. doi: 10.1016/j.janxdis.2008.01.008.
108. Kivi M, Eriksson MC, Hange D, Petersson EL, Vernmark K, Johansson B, et al. Internet-based therapy for mild to moderate depression in Swedish primary care: short term results from the PRIM-NET randomized controlled trial. Cognitive behaviour therapy. 2014;43(4):289-98. PMID: 24911260. doi: 10.1080/16506073.2014.921834.
109. Kleiboer A, Donker T, Seekles W, van Straten A, Riper H, Cuijpers P. A randomized controlled trial on the role of support in Internet-based problem solving therapy for depression and anxiety. Behaviour research and therapy. 2015 Sep;72(Journal Article):63-71. PMID: 26188373. doi: 10.1016/j.brat.2015.06.013.
110. Klein B, Richards JC, Austin DW. Efficacy of internet therapy for panic disorder. Journal of behavior therapy and experimental psychiatry. 2006 Sep;37(3):213-38. PMID: 16126161. doi: 10.1016/j.jbtep.2005.07.001.

111. Klein JP, Berger T, Schroder J, Spath C, Meyer B, Caspar F, et al. Effects of a Psychological Internet Intervention in the Treatment of Mild to Moderate Depressive Symptoms: Results of the EVIDENT Study, a Randomized Controlled Trial. Psychotherapy and psychosomatics. 2016;85(4):218-28. PMID: 27230863. doi: 10.1159/000445355.
112. Kok G, Burger H, Riper H, Cuijpers P, Dekker J, van Marwijk H, et al. The Three-Month Effect of Mobile Internet-Based Cognitive Therapy on the Course of Depressive Symptoms in Remitted Recurrently Depressed Patients: Results of a Randomized Controlled Trial. Psychotherapy and psychosomatics. 2015 Feb 21;84(2):90-9. PMID: 25721915. doi: 10.1159/000369469.
113. Kok RN, van Straten A, Beekman AT, Cuijpers P. Short-term effectiveness of web-based guided self-help for phobic outpatients: randomized controlled trial. Journal of medical Internet research. 2014 Sep 29;16(9):e226. PMID: 25266929. doi: 10.2196/jmir.3429.
114. Kruger JR, Kim P, Iyer V, Marko-Holguin M, Fogel J, DeFrino D, et al. Evaluation of protective and vulnerability factors for depression following an internet-based intervention to prevent depression in at-risk adolescents. International Journal of Mental Health Promotion. 2017 Apr;19(2):69-84. PMID: WOS:000400000000002. doi: 10.1080/14623730.2017.1308264.
115. Lappalainen P, Granlund A, Siltanen S, Ahonen S, Vitikainen M, Tolvanen A, et al. ACT Internet-based vs face-to-face? A randomized controlled trial of two ways to deliver Acceptance and Commitment Therapy for depressive symptoms: an 18-month follow-up. Behaviour research and therapy. 2014 Oct;61(Journal Article):43-54. PMID: 25127179. doi: 10.1016/j.brat.2014.07.006.
116. Lappalainen P, Langrial S, Oinas-Kukkonen H, Tolvanen A, Lappalainen R. Web-based acceptance and commitment therapy for depressive symptoms with minimal support: a randomized controlled trial. Behavior modification. 2015 Nov;39(6):805-34. PMID: 26253644. doi: 10.1177/0145445515598142.
117. Levin ME, Haeger JA, Pierce BG, Twohig MP. Web-Based Acceptance and Commitment Therapy for Mental Health Problems in College Students: A Randomized Controlled Trial. Behavior modification. 2017 Jan 1;41(1):141-62. PMID: 27440189. doi: 10.1177/0145445516659645.
118. Levin ME, Pistorello J, Seeley JR, Hayes SC. Feasibility of a prototype web-based acceptance and commitment therapy prevention program for college students. Journal of American college health : J of ACH. 2014;62(1):20-30. PMID: 24313693. doi: 10.1080/07448481.2013.843533.
119. Lintvedt OK, Griffiths KM, Sorensen K, Ostvik AR, Wang CE, Eisemann M, et al. Evaluating the effectiveness and efficacy of unguided internet-based self-help intervention for the prevention of depression: a randomized controlled trial. Clinical psychology & psychotherapy. 2013 Jan-Feb;20(1):10-27. PMID: 21887811. doi: 10.1002/cpp.770.
120. Low KGC, Swita; Lesser, Jill; Reinhalter, Katie; Martin, Rachel; Jones, Hannah; Winzelberg, Andy; Abascal, Liana; Taylor, C. Barr. Effectiveness of a computer-based interactive eating disorders prevention program at long-term follow-up. Eating disorders. 2006;14(1):17-30.
121. Mananes G, Vallejo MA. Usage and effectiveness of a fully automated, open-access, Spanish Web-based smoking cessation program: randomized controlled trial. Journal of medical Internet research. 2014 Apr 23;16(4):e111. PMID: 24760951. doi: 10.2196/jmir.3091.
122. McCall HC, Richardson CG, Helgadottir FD, Chen FS. Evaluating a Web-Based Social Anxiety Intervention Among University Students: Randomized Controlled Trial. Journal of medical Internet research. 2018 Mar 21;20(3):e91. PMID: 29563078. doi: 10.2196/jmir.8630.
123. Meyer B, Berger T, Caspar F, Beevers CG, Andersson G, Weiss M. Effectiveness of a novel integrative online treatment for depression (Deprexis): randomized controlled trial. Journal of medical Internet research. 2009 May 11;11(2):e15. PMID: 19632969. doi: 10.2196/jmir.1151.
124. Mira A, Breton-Lopez J, Garcia-Palacios A, Quero S, Banos RM, Botella C. An Internet-based program for depressive symptoms using human and automated support: a randomized controlled trial. Neuropsychiatr Dis Treat. 2017;13:987-1006. PMID: 28408833. doi: 10.2147/NDT.S130994.
125. Mogoaşe C, Brăilean A, David D. Can Concreteness Training Alone Reduce Depressive Symptoms? A Randomized Pilot Study Using an Internet-Delivered Protocol. Cognitive Therapy and Research. 2013;37(4):704-12. doi: 10.1007/s10608-012-9514-z.
126. Mohr DC, Duffecy J, Ho J, Kwasny M, Cai X, Burns MN, et al. A randomized controlled trial evaluating a manualized TeleCoaching protocol for improving adherence to a web-based intervention

for the treatment of depression. PloS one. 2013;8(8):e70086. PMID: 23990896. doi: 10.1371/journal.pone.0070086.
127. Montero-Marin J, Araya R, Perez-Yus MC, Mayoral F, Gili M, Botella C, et al. An Internet-Based Intervention for Depression in Primary Care in Spain: A Randomized Controlled Trial. Journal of medical Internet research. 2016 Aug 26;18(8):e231. PMID: 27565118. doi: 10.2196/jmir.5695.
128. Moritz S, Schilling L, Hauschildt M, Schroder J, Treszl A. A randomized controlled trial of internet-based therapy in depression. Behaviour research and therapy. 2012 Aug;50(7-8):513-21. PMID: 22677231. doi: 10.1016/j.brat.2012.04.006.
129. Morris RR, Schueller SM, Picard RW. Efficacy of a Web-based, crowdsourced peer-to-peer cognitive reappraisal platform for depression: randomized controlled trial. Journal of medical Internet research. 2015 Mar 30;17(3):e72. PMID: 25835472. doi: 10.2196/jmir.4167.
130. Munoz RF, Barrera AZ, Delucchi K, Penilla C, Torres LD, Perez-Stable EJ. International Spanish/English Internet smoking cessation trial yields 20% abstinence rates at 1 year. Nicotine & tobacco research : official journal of the Society for Research on Nicotine and Tobacco. 2009 Sep;11(9):1025-34. PMID: 19640833. doi: 10.1093/ntr/ntp090.
131. Musiat P, Conrod P, Treasure J, Tylee A, Williams C, Schmidt U. Targeted prevention of common mental health disorders in university students: randomised controlled trial of a transdiagnostic trait-focused web-based intervention. PloS one. 2014;9(4):e93621. PMID: 24736388. doi: 10.1371/journal.pone.0093621.
132. Newby JM, Mackenzie A, Williams AD, McIntyre K, Watts S, Wong N, et al. Internet cognitive behavioural therapy for mixed anxiety and depression: a randomized controlled trial and evidence of effectiveness in primary care. Psychological medicine. 2013 Dec;43(12):2635-48. PMID: 23419552. doi: 10.1017/S0033291713000111.
133. Nystrom MBT, Stenling A, Sjostrom E, Neely G, Lindner P, Hassmen P, et al. Behavioral activation versus physical activity via the internet: A randomized controlled trial. Journal of affective disorders. 2017 Jun;215:85-93. PMID: 28319696. doi: 10.1016/j.jad.2017.03.018.
134. Oromendia P, Orrego J, Bonillo A, Molinuevo B. Internet-based self-help treatment for panic disorder: a randomized controlled trial comparing mandatory versus optional complementary psychological support. Cognitive behaviour therapy. 2016 Jun;45(4):270-86. PMID: 27007256. doi: 10.1080/16506073.2016.1163615.
135. Paxling B, Almlov J, Dahlin M, Carlbring P, Breitholtz E, Eriksson T, et al. Guided internet-delivered cognitive behavior therapy for generalized anxiety disorder: a randomized controlled trial. Cognitive behaviour therapy. 2011;40(3):159-73. PMID: 21770848. doi: 10.1080/16506073.2011.576699.
136. Perini S, Titov N, Andrews G. Clinician-assisted Internet-based treatment is effective for depression: randomized controlled trial. The Australian and New Zealand journal of psychiatry. 2009 Jun;43(6):571-8. PMID: 19440890. doi: 10.1080/00048670902873722.
137. Phillips R, Schneider J, Molosankwe I, Leese M, Foroushani PS, Grime P, et al. Randomized controlled trial of computerized cognitive behavioural therapy for depressive symptoms: effectiveness and costs of a workplace intervention. Psychological medicine. 2014 Mar;44(4):741-52. PMID: 23795621. doi: 10.1017/S0033291713001323.
138. Pisinger C, Jorgensen MM, Moller NE, Dossing M, Jorgensen T. A cluster randomized trial in general practice with referral to a group-based or an Internet-based smoking cessation programme. J Public Health (Oxf). 2010 Mar;32(1):62-70. PMID: 19617300. doi: 10.1093/pubmed/fdp072.
139. Postel MG, de Haan HA, ter Huurne ED, Becker ES, de Jong CA. Effectiveness of a web-based intervention for problem drinkers and reasons for dropout: randomized controlled trial. Journal of medical Internet research. 2010 Dec 16;12(4):e68. PMID: 21163776. doi: 10.2196/jmir.1642.
140. Pots WT, Fledderus M, Meulenbeek PA, ten Klooster PM, Schreurs KM, Bohlmeijer ET. Acceptance and commitment therapy as a web-based intervention for depressive symptoms: randomised controlled trial. The British journal of psychiatry : the journal of mental science. 2016 Jan;208(1):69-77. PMID: 26250745. doi: 10.1192/bjp.bp.114.146068.
141. Powell J, Hamborg T, Stallard N, Burls A, McSorley J, Bennett K, et al. Effectiveness of a web-based cognitive-behavioral tool to improve mental well-being in the general population: randomized

controlled trial. Journal of medical Internet research. 2012 Dec 31;15(1):e2. PMID: 23302475. doi: 10.2196/jmir.2240.
142. Radhu N, Daskalakis ZJ, Arpin-Cribbie CA, Irvine J, Ritvo P. Evaluating a Web-based cognitive-behavioral therapy for maladaptive perfectionism in university students. Journal of American college health : J of ACH. 2012;60(5):357-66. PMID: 22686358. doi: 10.1080/07448481.2011.630703.
143. Richards D, Timulak L, O'Brien E, Hayes C, Vigano N, Sharry J, et al. A randomized controlled trial of an internet-delivered treatment: Its potential as a low-intensity community intervention for adults with symptoms of depression. Behaviour research and therapy. 2015 Dec;75(Journal Article):20-31. PMID: 26523885. doi: 10.1016/j.brat.2015.10.005.
144. Richards JC, Klein B, Austin DW. Internet cognitive behavioural therapy for panic disorder: Does the inclusion of stress management information improve end-state functioning? Clinical Psychologist. 2006;10(1):2-15. doi: 10.1080/13284200500378795.
145. Rickhi B, Kania-Richmond A, Moritz S, Cohen J, Paccagnan P, Dennis C, et al. Evaluation of a spirituality informed e-mental health tool as an intervention for major depressive disorder in adolescents and young adults - a randomized controlled pilot trial. BMC complementary and alternative medicine. 2015 Dec 24;15(Journal Article):450. PMID: 26702639. doi: 10.1186/s12906-015-0968-x.
146. Riper H, Kramer J, Smit F, Conijn B, Schippers G, Cuijpers P. Web-based self-help for problem drinkers: a pragmatic randomized trial. Addiction (Abingdon, England). 2008 Feb;103(2):218-27. PMID: 18199300. doi: 10.1111/j.1360-0443.2007.02063.x.
147. Robinson E, Titov N, Andrews G, McIntyre K, Schwencke G, Solley K. Internet treatment for generalized anxiety disorder: a randomized controlled trial comparing clinician vs. technician assistance. PloS one. 2010 Jun 3;5(6):e10942. PMID: 20532167. doi: 10.1371/journal.pone.0010942.
148. Rollman BL, Herbeck Belnap B, Abebe KZ, Spring MB, Rotondi AJ, Rothenberger SD, et al. Effectiveness of Online Collaborative Care for Treating Mood and Anxiety Disorders in Primary Care: A Randomized Clinical Trial. JAMA psychiatry. 2018 Jan 1;75(1):56-64. PMID: 29117275. doi: 10.1001/jamapsychiatry.2017.3379.
149. Rooke S, Copeland J, Norberg M, Hine D, McCambridge J. Effectiveness of a self-guided web-based cannabis treatment program: randomized controlled trial. Journal of medical Internet research. 2013 Feb 15;15(2):e26. PMID: 23470329. doi: 10.2196/jmir.2256.
150. Rosmarin DH, Pargament KI, Pirutinsky S, Mahoney A. A randomized controlled evaluation of a spiritually integrated treatment for subclinical anxiety in the Jewish community, delivered via the Internet. Journal of anxiety disorders. 2010 Oct;24(7):799-808. PMID: 20591614. doi: 10.1016/j.janxdis.2010.05.014.
151. Rosso IM, Killgore WD, Olson EA, Webb CA, Fukunaga R, Auerbach RP, et al. Internet-based cognitive behavior therapy for major depressive disorder: A randomized controlled trial. Depress Anxiety. 2017 Mar;34(3):236-45. PMID: 28009467. doi: 10.1002/da.22590.
152. Ruwaard J, Broeksteeg J, Schrieken B, Emmelkamp P, Lange A. Web-based therapist-assisted cognitive behavioral treatment of panic symptoms: a randomized controlled trial with a three-year follow-up. Journal of anxiety disorders. 2010 May;24(4):387-96. PMID: 20227241. doi: 10.1016/j.janxdis.2010.01.010.
153. Ruwaard J, Lange A, Broeksteeg J, Renteria-Agirre A, Schrieken B, Dolan CV, et al. Online cognitive-behavioural treatment of bulimic symptoms: a randomized controlled trial. Clinical psychology & psychotherapy. 2013 Jul-Aug;20(4):308-18. PMID: 22298417. doi: 10.1002/cpp.1767.
154. Ruwaard J, Schrieken B, Schrijver M, Broeksteeg J, Dekker J, Vermeulen H, et al. Standardized web-based cognitive behavioural therapy of mild to moderate depression: a randomized controlled trial with a long-term follow-up. Cognitive behaviour therapy. 2009;38(4):206-21. PMID: 19221919. doi: 10.1080/16506070802408086.
155. Saekow J, Jones M, Gibbs E, Jacobi C, Fitzsimmons-Craft EE, Wilfley D, et al. StudentBodies-eating disorders: A randomized controlled trial of a coached online intervention for subclinical eating disorders. Internet Interventions. 2015;2(4):419-28. doi: 10.1016/j.invent.2015.10.004.
156. Sanchez R, Brown E, Kocher K, DeRosier M. Improving Children's Mental Health with a Digital Social Skills Development Game: A Randomized Controlled Efficacy Trial of Adventures aboard the

S.S. GRIN. Games for health journal. 2017 Feb;6(1):19-27. PMID: 28051877. doi: 10.1089/g4h.2015.0108.
157. Sanchez-Ortiz VC, Munro C, Stahl D, House J, Startup H, Treasure J, et al. A randomized controlled trial of internet-based cognitive-behavioural therapy for bulimia nervosa or related disorders in a student population. Psychological medicine. 2011 Feb;41(2):407-17. PMID: 20406523. doi: 10.1017/S0033291710000711.
158. Schaub M, Sullivan R, Haug S, Stark L. Web-based cognitive behavioral self-help intervention to reduce cocaine consumption in problematic cocaine users: randomized controlled trial. Journal of medical Internet research. 2012 Nov 28;14(6):e166. PMID: 23192752. doi: 10.2196/jmir.2244.
159. Schaub MP, Wenger A, Berg O, Beck T, Stark L, Buehler E, et al. A Web-Based Self-Help Intervention With and Without Chat Counseling to Reduce Cannabis Use in Problematic Cannabis Users: Three-Arm Randomized Controlled Trial. Journal of medical Internet research. 2015 Oct 13;17(10):e232. PMID: 26462848. doi: 10.2196/jmir.4860.
160. Schroder J, Jelinek L, Moritz S. A randomized controlled trial of a transdiagnostic Internet intervention for individuals with panic and phobias - One size fits all. Journal of behavior therapy and experimental psychiatry. 2017 Mar;54:17-24. PMID: 27227651. doi: 10.1016/j.jbtep.2016.05.002.
161. Schueller SMP-S, E. J.; Munoz, R. F. A Mood Management Intervention in an Internet Stop Smoking Randomized Controlled Trial Does Not Prevent Depression: A Cautionary Tale. Clinical psychological science : a journal of the Association for Psychological Science. 2013;1(4):401-12. doi: 10.1177/2167702613484717 [doi].
162. Schulz A, Stolz T, Vincent A, Krieger T, Andersson G, Berger T. A sorrow shared is a sorrow halved? A three-arm randomized controlled trial comparing internet-based clinician-guided individual versus group treatment for social anxiety disorder. Behaviour research and therapy. 2016 Sep;84:14-26. PMID: 27423374. doi: 10.1016/j.brat.2016.07.001.
163. Seidman DF, Westmaas JL, Goldband S, Rabius V, Katkin ES, Pike KJ, et al. Randomized controlled trial of an interactive internet smoking cessation program with long-term follow-up. Ann Behav Med. 2010 Feb;39(1):48-60. PMID: 20177844. doi: 10.1007/s12160-010-9167-7.
164. Sethi S. Treating Youth Depression and Anxiety: A Randomised Controlled Trial Examining the Efficacy of Computerised versus Face-to-face Cognitive Behaviour Therapy. Aust Psychol. 2013 Aug;48(4):249-57. PMID: WOS:000321626500003. doi: 10.1111/ap.12006.
165. Sethi S, Campbell AJ, Ellis LA. The Use of Computerized Self-Help Packages to Treat Adolescent Depression and Anxiety. Journal of Technology in Human Services. 2010;28(3):144-60. doi: 10.1080/15228835.2010.508317.
166. Sheeber LB, Feil EG, Seeley JR, Leve C, Gau JM, Davis B, et al. Mom-net: Evaluation of an internet-facilitated cognitive behavioral intervention for low-income depressed mothers. Journal of consulting and clinical psychology. 2017 Apr;85(4):355-66. PMID: 28333536. doi: 10.1037/ccp0000175.
167. Sheeber LB, Seeley JR, Feil EG, Davis B, Sorensen E, Kosty DB, et al. Development and pilot evaluation of an Internet-facilitated cognitive-behavioral intervention for maternal depression. Journal of consulting and clinical psychology. 2012 Oct;80(5):739-49. PMID: 22663903. doi: 10.1037/a0028820.
168. Silfvernagel K, Carlbring P, Kabo J, Edstrom S, Eriksson J, Manson L, et al. Individually tailored internet-based treatment for young adults and adults with panic attacks: randomized controlled trial. Journal of medical Internet research. 2012 Jun 26;14(3):e65. PMID: 22732098. doi: 10.2196/jmir.1853.
169. Spek V, Nyklicek I, Smits N, Cuijpers P, Riper H, Keyzer J, et al. Internet-based cognitive behavioural therapy for subthreshold depression in people over 50 years old: a randomized controlled clinical trial. Psychological medicine. 2007 Dec;37(12):1797-806. PMID: 17466110. doi: 10.1017/S0033291707000542.
170. Stice E, Rohde P, Durant S, Shaw H. A preliminary trial of a prototype Internet dissonance-based eating disorder prevention program for young women with body image concerns. Journal of consulting and clinical psychology. 2012 Oct;80(5):907-16. PMID: 22506791. doi: 10.1037/a0028016.

171. Stice E, Rohde P, Shaw H, Gau JM. Clinician-led, peer-led, and internet-delivered dissonance-based eating disorder prevention programs: Acute effectiveness of these delivery modalities. Journal of consulting and clinical psychology. 2017 Sep;85(9):883-95. PMID: 28425735. doi: 10.1037/ccp0000211.
172. Stolz T, Schulz A, Krieger T, Vincent A, Urech A, Moser C, et al. A mobile app for social anxiety disorder: A three-arm randomized controlled trial comparing mobile and PC-based guided self-help interventions. Journal of consulting and clinical psychology. 2018 Jun;86(6):493-504. PMID: 29781648. doi: 10.1037/ccp0000301.
173. Strandskov SW, Ghaderi A, Andersson H, Parmskog N, Hjort E, Warn AS, et al. Effects of Tailored and ACT-Influenced Internet-Based CBT for Eating Disorders and the Relation Between Knowledge Acquisition and Outcome: A Randomized Controlled Trial. Behavior therapy. 2017 Sep;48(5):624-37. PMID: 28711113. doi: 10.1016/j.beth.2017.02.002.
174. Strom M, Uckelstam CJ, Andersson G, Hassmen P, Umefjord G, Carlbring P. Internet-delivered therapist-guided physical activity for mild to moderate depression: a randomized controlled trial. PeerJ. 2013;1(Journal Article):e178. PMID: 24109561. doi: 10.7717/peerj.178.
175. Sundstrom C, Gajecki M, Johansson M, Blankers M, Sinadinovic K, Stenlund-Gens E, et al. Guided and Unguided Internet-Based Treatment for Problematic Alcohol Use - A Randomized Controlled Pilot Trial. PloS one. 2016;11(7):e0157817. PMID: 27383389. doi: 10.1371/journal.pone.0157817.
176. Tait RJ, McKetin R, Kay-Lambkin F, Carron-Arthur B, Bennett A, Bennett K, et al. A Web-Based Intervention for Users of Amphetamine-Type Stimulants: 3-Month Outcomes of a Randomized Controlled Trial. JMIR mental health. 2014 Jul-Dec;1(1):e1. PMID: 26543901. doi: 10.2196/mental.3278.
177. Taylor CB, Bryson S, Luce KH, Cunning D, Doyle AC, Abascal LB, et al. Prevention of eating disorders in at-risk college-age women. Archives of general psychiatry. 2006 Aug;63(8):881-8. PMID: 16894064. doi: 10.1001/archpsyc.63.8.881.
178. Taylor CB, Kass AE, Trockel M, Cunning D, Weisman H, Bailey J, et al. Reducing eating disorder onset in a very high risk sample with significant comorbid depression: A randomized controlled trial. Journal of consulting and clinical psychology. 2016 May;84(5):402-14. PMID: 26795936. doi: 10.1037/ccp0000077.
179. ter Huurne ED, de Haan HA, Postel MG, van der Palen J, VanDerNagel JE, DeJong CA. Web-Based Cognitive Behavioral Therapy for Female Patients With Eating Disorders: Randomized Controlled Trial. Journal of medical Internet research. 2015 Jun 18;17(6):e152. PMID: 26088580. doi: 10.2196/jmir.3946.
180. Terides MD, Dear BF, Fogliati VJ, Gandy M, Karin E, Jones MP, et al. Increased skills usage statistically mediates symptom reduction in self-guided internet-delivered cognitive-behavioural therapy for depression and anxiety: a randomised controlled trial. Cognitive behaviour therapy. 2018 Jan;47(1):43-61. PMID: 28724338. doi: 10.1080/16506073.2017.1347195.
181. Tiburcio M, Lara MA, Martinez N, Fernandez M, Aguilar A. Web-Based Intervention to Reduce Substance Abuse and Depression: A Three Arm Randomized Trial in Mexico. Substance use & misuse. 2018 Nov 10;53(13):2220-31. PMID: 29768070. doi: 10.1080/10826084.2018.1467452.
182. Tillfors M, Andersson G, Ekselius L, Furmark T, Lewenhaupt S, Karlsson A, et al. A randomized trial of Internet-delivered treatment for social anxiety disorder in high school students. Cognitive behaviour therapy. 2011;40(2):147-57. PMID: 25155815. doi: 10.1080/16506073.2011.555486.
183. Tillfors M, Carlbring P, Furmark T, Lewenhaupt S, Spak M, Eriksson A, et al. Treating university students with social phobia and public speaking fears: Internet delivered self-help with or without live group exposure sessions. Depress Anxiety. 2008;25(8):708-17. PMID: 18729147. doi: 10.1002/da.20416.
184. Titov N, Andrews G, Choi I, Schwencke G, Johnston L. Randomized controlled trial of web-based treatment of social phobia without clinician guidance. Aust Nz J Psychiat. 2009;43(10):913-9. PMID: WOS:000271406200004. doi: Doi 10.1080/00048670903179160.

185. Titov N, Andrews G, Choi I, Schwencke G, Mahoney A. Shyness 3: randomized controlled trial of guided versus unguided Internet-based CBT for social phobia. The Australian and New Zealand journal of psychiatry. 2008 Dec;42(12):1030-40. PMID: 19016091. doi: 10.1080/00048670802512107.
186. Titov N, Andrews G, Davies M, McIntyre K, Robinson E, Solley K. Internet treatment for depression: a randomized controlled trial comparing clinician vs. technician assistance. PloS one. 2010 Jun 8;5(6):e10939. PMID: 20544030. doi: 10.1371/journal.pone.0010939.
187. Titov N, Andrews G, Johnston L, Robinson E, Spence J. Transdiagnostic Internet treatment for anxiety disorders: A randomized controlled trial. Behaviour research and therapy. 2010 Sep;48(9):890-9. PMID: 20561606. doi: 10.1016/j.brat.2010.05.014.
188. Titov N, Andrews G, Robinson E, Schwencke G, Johnston L, Solley K, et al. Clinician-assisted Internet-based treatment is effective for generalized anxiety disorder: randomized controlled trial. Aust Nz J Psychiat. 2009;43(10):905-12. PMID: WOS:000271406200003. doi: Doi 10.1080/00048670903179269.
189. Titov N, Andrews G, Schwencke G. Shyness 2: treating social phobia online: replication and extension. The Australian and New Zealand journal of psychiatry. 2008 Jul;42(7):595-605. PMID: 18612863. doi: 10.1080/00048670802119820.
190. Titov N, Andrews G, Schwencke G, Drobny J, Einstein D. Shyness 1: distance treatment of social phobia over the Internet. The Australian and New Zealand journal of psychiatry. 2008 Jul;42(7):585-94. PMID: 18612862. doi: 10.1080/00048670802119762.
191. Titov N, Andrews G, Schwencke G, Robinson E, Peters L, Spence J. Randomized controlled trial of Internet cognitive behavioural treatment for social phobia with and without motivational enhancement strategies. The Australian and New Zealand journal of psychiatry. 2010 Oct;44(10):938-45. PMID: 20932208. doi: 10.3109/00048674.2010.493859.
192. Titov N, Andrews G, Schwencke G, Solley K, Johnston L, Robinson E. An RCT comparing effect of two types of support on severity of symptoms for people completing Internet-based cognitive behaviour therapy for social phobia. Aust Nz J Psychiat. 2009;43(10):920-6. PMID: WOS:000271406200005. doi: Doi 10.1080/00048670903179228.
193. Titov N, Dear BF, Ali S, Zou JB, Lorian CN, Johnston L, et al. Clinical and cost-effectiveness of therapist-guided internet-delivered cognitive behavior therapy for older adults with symptoms of depression: a randomized controlled trial. Behavior therapy. 2015 Mar;46(2):193-205. PMID: 25645168. doi: 10.1016/j.beth.2014.09.008.
194. Titov N, Dear BF, Johnston L, Lorian C, Zou J, Wootton B, et al. Improving adherence and clinical outcomes in self-guided internet treatment for anxiety and depression: randomised controlled trial. PloS one. 2013;8(7):e62873. PMID: 23843932. doi: 10.1371/journal.pone.0062873.
195. Titov N, Dear BF, Schwencke G, Andrews G, Johnston L, Craske MG, et al. Transdiagnostic internet treatment for anxiety and depression: a randomised controlled trial. Behaviour research and therapy. 2011 Aug;49(8):441-52. PMID: 21679925. doi: 10.1016/j.brat.2011.03.007.
196. Titov N, Dear BF, Staples LG, Terides MD, Karin E, Sheehan J, et al. Disorder-specific versus transdiagnostic and clinician-guided versus self-guided treatment for major depressive disorder and comorbid anxiety disorders: A randomized controlled trial. Journal of anxiety disorders. 2015 Oct;35(Journal Article):88-102. PMID: 26422822. doi: 10.1016/j.janxdis.2015.08.002.
197. Titov N, Fogliati VJ, Staples LG, Gandy M, Johnston L, Wootton B, et al. Treating anxiety and depression in older adults: randomised controlled trial comparing guided v. self-guided internet-delivered cognitive-behavioural therapy. BJPsych open. 2016 Jan;2(1):50-8. PMID: 27703754. doi: 10.1192/bjpo.bp.115.002139.
198. Tulbure BT, Szentagotai A, David O, Stefan S, Mansson KN, David D, et al. Internet-delivered cognitive-behavioral therapy for social anxiety disorder in Romania: a randomized controlled trial. PloS one. 2015;10(5):e0123997. PMID: 25938241. doi: 10.1371/journal.pone.0123997.
199. Twomey C, O'Reilly G, Byrne M, Bury M, White A, Kissane S, et al. A randomized controlled trial of the computerized CBT programme, MoodGYM, for public mental health service users waiting for interventions. The British journal of clinical psychology. 2014 Nov;53(4):433-50. PMID: 24831119. doi: 10.1111/bjc.12055.

200. Unlu Ince B, Cuijpers P, van 't Hof E, van Ballegooijen W, Christensen H, Riper H. Internet-based, culturally sensitive, problem-solving therapy for Turkish migrants with depression: randomized controlled trial. Journal of medical Internet research. 2013 Oct 11;15(10):e227. PMID: 24121307. doi: 10.2196/jmir.2853.
201. van Ballegooijen W, Riper H, Klein B, Ebert DD, Kramer J, Meulenbeek P, et al. An Internet-based guided self-help intervention for panic symptoms: randomized controlled trial. Journal of medical Internet research. 2013 Jul 29;15(7):e154. PMID: 23896222. doi: 10.2196/jmir.2362.
202. van der Zweerde T, van Straten A, Effting M, Kyle SD, Lancee J. Does online insomnia treatment reduce depressive symptoms? A randomized controlled trial in individuals with both insomnia and depressive symptoms. Psychological medicine. 2018 May 11:1-9. PMID: 29747706. doi: 10.1017/S0033291718001149.
203. van Straten A, Cuijpers P, Smits N. Effectiveness of a web-based self-help intervention for symptoms of depression, anxiety, and stress: randomized controlled trial. Journal of medical Internet research. 2008 Mar 25;10(1):e7. PMID: 18364344. doi: 10.2196/jmir.954.
204. Vernmark K, Lenndin J, Bjarehed J, Carlsson M, Karlsson J, Oberg J, et al. Internet administered guided self-help versus individualized e-mail therapy: A randomized trial of two versions of CBT for major depression. Behaviour research and therapy. 2010 May;48(5):368-76. PMID: 20152960. doi: 10.1016/j.brat.2010.01.005.
205. Wagner B, Horn AB, Maercker A. Internet-based versus face-to-face cognitive-behavioral intervention for depression: a randomized controlled non-inferiority trial. Journal of affective disorders. 2014 Jan;152-154(Journal Article):113-21. PMID: 23886401. doi: 10.1016/j.jad.2013.06.032.
206. Wagner G, Penelo E, Wanner C, Gwinner P, Trofaier ML, Imgart H, et al. Internet-delivered cognitive-behavioural therapy v. conventional guided self-help for bulimia nervosa: long-term evaluation of a randomised controlled trial. The British journal of psychiatry : the journal of mental science. 2013 Feb;202(2):135-41. PMID: 23222037. doi: 10.1192/bjp.bp.111.098582.
207. Warmerdam L, van Straten A, Twisk J, Riper H, Cuijpers P. Internet-based treatment for adults with depressive symptoms: randomized controlled trial. Journal of medical Internet research. 2008 Nov 20;10(4):e44. PMID: 19033149. doi: 10.2196/jmir.1094.
208. Watts S, Mackenzie A, Thomas C, Griskaitis A, Mewton L, Williams A, et al. CBT for depression: a pilot RCT comparing mobile phone vs. computer. BMC psychiatry. 2013 Feb 7;13(Journal Article):49. PMID: 23391304. doi: 10.1186/1471-244X-13-49.
209. White MA, Ivezaj V, Grilo CM. Evaluation of a web-based cognitive behavioral smoking cessation treatment for overweight/obese smokers. Journal of health psychology. 2017 Apr 1:1359105317701560. PMID: 28810442. doi: 10.1177/1359105317701560.
210. Wilksch SM, O'Shea A, Taylor CB, Wilfley D, Jacobi C, Wade TD. Online prevention of disordered eating in at-risk young-adult women: a two-country pragmatic randomized controlled trial. Psychological medicine. 2018 Sep;48(12):2034-44. PMID: 29233196. doi: 10.1017/S0033291717003567.
211. Williams AD, Blackwell SE, Mackenzie A, Holmes EA, Andrews G. Combining imagination and reason in the treatment of depression: a randomized controlled trial of internet-based cognitive-bias modification and internet-CBT for depression. Journal of consulting and clinical psychology. 2013 Oct;81(5):793-9. PMID: 23750459. doi: 10.1037/a0033247.
212. Williams AD, O'Moore K, Blackwell SE, Smith J, Holmes EA, Andrews G. Positive imagery cognitive bias modification (CBM) and internet-based cognitive behavioral therapy (iCBT): a randomized controlled trial. Journal of affective disorders. 2015 Jun 1;178(Journal Article):131-41. PMID: 25805405. doi: 10.1016/j.jad.2015.02.026.
213. Wims E, Titov N, Andrews G, Choi I. Clinician-assisted Internet-based treatment is effective for panic: A randomized controlled trial. The Australian and New Zealand journal of psychiatry. 2010 Jul;44(7):599-607. PMID: 20560847. doi: 10.3109/00048671003614171.
214. Yeung A, Wang F, Feng F, Zhang J, Cooper A, Hong L, et al. Outcomes of an online computerized cognitive behavioral treatment program for treating chinese patients with depression:

A pilot study. Asian journal of psychiatry. 2017 Nov 11. PMID: 29146042. doi: 10.1016/j.ajp.2017.11.007.
215. Zagorscak P, Heinrich M, Sommer D, Wagner B, Knaevelsrud C. Benefits of Individualized Feedback in Internet-Based Interventions for Depression: A Randomized Controlled Trial. Psychotherapy and psychosomatics. 2018;87(1):32-45. PMID: 29306945. doi: 10.1159/000481515.
216. Zwerenz R, Becker J, Johansson R, Frederick RJ, Andersson G, Beutel ME. Transdiagnostic, Psychodynamic Web-Based Self-Help Intervention Following Inpatient Psychotherapy: Results of a Feasibility Study and Randomized Controlled Trial. JMIR mental health. 2017 Oct 16;4(4):e41. PMID: 29038094. doi: 10.2196/mental.7889.

Search String:

We used the following search Strings: 1) Web of Science: TS= ((online OR internet OR web-based OR mobile) AND (treatment OR psychotherapy OR therapy OR self-help OR prevention OR intervention) AND (depression OR depressive OR anxiety OR phobia OR phobic OR eating disorder OR disordered eating OR anorexia OR anorexic OR bulimia OR bulimic OR binge eating OR substance abuse OR substance related disorder OR alcohol OR nicotine OR cannabis) AND (RCT OR randomized OR randomised)) Timespan: 2006-2018; 2) Pubmed: ((((online[Title/Abstract] OR internet[Title/Abstract] OR web-based[Title/Abstract] OR mobile[Title/Abstract]) AND (treatment[Title/Abstract] OR psychotherapy[Title/Abstract] OR therapy[Title/Abstract] OR self-help[Title/Abstract] OR prevention[Title/Abstract] OR intervention[Title/Abstract])) AND (depression[Title/Abstract] OR depressive[Title/Abstract] OR anxiety[Title/Abstract] OR phobia[Title/Abstract] OR phobic[Title/Abstract] OR eating disorder[Title/Abstract] OR disordered eating[Title/Abstract] OR anorexia[Title/Abstract] OR anorexic[Title/Abstract] OR bulimia[Title/Abstract] OR bulimic[Title/Abstract] OR binge eating[Title/Abstract] OR substance abuse[Title/Abstract] OR substance-related disorder[Title/Abstract] OR alcohol[Title/Abstract] OR nicotine[Title/Abstract] OR cannabis[Title/Abstract])) AND (RCT[Title/Abstract] OR randomized[Title/Abstract] OR randomised[Title/Abstract])) AND ("2006"[PDAT] : "2018"[PDAT]).
